# Associations of variants In the hexokinase 1 and interleukin 18 receptor regions with oxyhemoglobin saturation during sleep

**DOI:** 10.1371/journal.pgen.1007739

**Published:** 2019-04-16

**Authors:** Brian E. Cade, Han Chen, Adrienne M. Stilp, Tin Louie, Sonia Ancoli-Israel, Raanan Arens, Richard Barfield, Jennifer E. Below, Jianwen Cai, Matthew P. Conomos, Daniel S. Evans, Alexis C. Frazier-Wood, Sina A. Gharib, Kevin J. Gleason, Daniel J. Gottlieb, David R. Hillman, W. Craig Johnson, David J. Lederer, Jiwon Lee, Jose S. Loredo, Hao Mei, Sutapa Mukherjee, Sanjay R. Patel, Wendy S. Post, Shaun M. Purcell, Alberto R. Ramos, Kathryn J. Reid, Ken Rice, Neomi A. Shah, Tamar Sofer, Kent D. Taylor, Timothy A. Thornton, Heming Wang, Kristine Yaffe, Phyllis C. Zee, Craig L. Hanis, Lyle J. Palmer, Jerome I. Rotter, Katie L. Stone, Gregory J. Tranah, James G. Wilson, Shamil R. Sunyaev, Cathy C. Laurie, Xiaofeng Zhu, Richa Saxena, Xihong Lin, Susan Redline

**Affiliations:** 1 Division of Sleep and Circadian Disorders, Brigham and Women’s Hospital, Boston, MA, United States of America; 2 Division of Sleep Medicine, Harvard Medical School, Boston, MA, United States of America; 3 Program in Medical and Population Genetics, Broad Institute, Cambridge, MA, United States of America; 4 Human Genetics Center, Department of Epidemiology, Human Genetics and Environmental Sciences, School of Public Health, The University of Texas Health Science Center at Houston, Houston, TX United States of America; 5 Center for Precision Health, School of Public Health and School of Biomedical Informatics, The University of Texas Health Science Center at Houston, Houston, TX United States of America; 6 Department of Biostatistics, University of Washington, Seattle, WA United States of America; 7 Department of Psychiatry, University of California, San Diego, CA, United States of America; 8 The Children’s Hospital at Montefiore, Division of Respiratory and Sleep Medicine, Albert Einstein College of Medicine, Bronx, NY, United States of America; 9 Department of Biostatistics, Harvard T.H. Chan School of Public Health, Boston, MA, United States of America; 10 Vanderbilt Genetics Institute, Vanderbilt University Medical Center, Nashville, TN, United States of America; 11 Department of Biostatistics, Gillings School of Global Public Health, University of North Carolina, Chapel Hill, NC, United States of America; 12 California Pacific Medical Center Research Institute, San Francisco, CA, United States of America; 13 USDA/ARS Children's Nutrition Research Center, Baylor College of Medicine, Houston, TX, United States of America; 14 Computational Medicine Core, Center for Lung Biology, UW Medicine Sleep Center, Division of Pulmonary, Critical Care and Sleep Medicine, University of Washington, Seattle WA, United States of America; 15 Department of Public Health Sciences, University of Chicago, Chicago, IL, United States of America; 16 VA Boston Healthcare System, Boston, MA, United States of America; 17 Department of Pulmonary Physiology and Sleep Medicine, Sir Charles Gairdner Hospital, Perth, Western Australia, Australia; 18 Departments of Medicine and Epidemiology, Columbia University, New York, NY, United States of America; 19 Division of Pulmonary Critical Care and Sleep Medicine, Department of Medicine, UC San Diego School of Medicine, La Jolla, CA, United States of America; 20 Department of Data Science, University of Mississippi Medical Center, Jackson, MS, United States of America; 21 Sleep Health Service, Respiratory and Sleep Services, Southern Adelaide Local Health Network, Adelaide, South Australia; 22 Adelaide Institute for Sleep Health, Flinders University, Adelaide, South Australia; 23 Division of Pulmonary, Allergy, and Critical Care Medicine, University of Pittsburgh, Pittsburgh, PA, United States of America; 24 Division of Cardiology, Johns Hopkins University, Baltimore, MD, United States of America; 25 Department of Neurology, University of Miami Miller School of Medicine, Miami, FL, United States of America; 26 Department of Neurology, Center for Circadian and Sleep Medicine, Northwestern University Feinberg School of Medicine, Chicago, IL, United States of America; 27 Division of Pulmonary, Critical Care and Sleep Medicine, Icahn School of Medicine at Mount Sinai, New York, NY, United States of America; 28 The Institute for Translational Genomics and Population Sciences, Departments of Pediatrics and Medicine, LABioMed at Harbor-UCLA Medical Center, Torrance, CA, United States of America; 29 Department of Psychiatry, Neurology, and Epidemiology and Biostatistics, University of California at San Francisco, San Francisco, CA, United States of America; 30 San Francisco VA Medical Center, San Francisco, CA, United States of America; 31 School of Public Health, University of Adelaide, South Australia, Australia; 32 Department of Physiology and Biophysics, University of Mississippi Medical Center, Jackson MS, United States of America; 33 Division of Genetics, Brigham and Women's Hospital, Boston, MA, United States of America; 34 Division of Medical Sciences, Harvard Medical School, Boston, MA, United States of America; 35 Department of Population and Quantitative Health Sciences, Case Western Reserve University, Cleveland, OH, United States of America; 36 Center for Genomic Medicine and Department of Anesthesia, Pain, and Critical Care Medicine, Massachusetts General Hospital, Boston, MA, United States of America; 37 Division of Pulmonary, Critical Care, and Sleep Medicine, Beth Israel Deaconess Medical Center, Boston, MA, United States of America; Oklahoma Medical Research Foundation, UNITED STATES

## Abstract

Sleep disordered breathing (SDB)-related overnight hypoxemia is associated with cardiometabolic disease and other comorbidities. Understanding the genetic bases for variations in nocturnal hypoxemia may help understand mechanisms influencing oxygenation and SDB-related mortality. We conducted genome-wide association tests across 10 cohorts and 4 populations to identify genetic variants associated with three correlated measures of overnight oxyhemoglobin saturation: average and minimum oxyhemoglobin saturation during sleep and the percent of sleep with oxyhemoglobin saturation under 90%. The discovery sample consisted of 8,326 individuals. Variants with p < 1 × 10^−6^ were analyzed in a replication group of 14,410 individuals. We identified 3 significantly associated regions, including 2 regions in multi-ethnic analyses (2q12, 10q22). SNPs in the 2q12 region associated with minimum SpO_2_ (rs78136548 p = 2.70 × 10^−10^). SNPs at 10q22 were associated with all three traits including average SpO_2_ (rs72805692 p = 4.58 × 10^−8^). SNPs in both regions were associated in over 20,000 individuals and are supported by prior associations or functional evidence. Four additional significant regions were detected in secondary sex-stratified and combined discovery and replication analyses, including a region overlapping Reelin, a known marker of respiratory complex neurons.These are the first genome-wide significant findings reported for oxyhemoglobin saturation during sleep, a phenotype of high clinical interest. Our replicated associations with *HK1* and *IL18R1* suggest that variants in inflammatory pathways, such as the biologically-plausible *NLRP3* inflammasome, may contribute to nocturnal hypoxemia.

## Author summary

Variation in oxyhemoglobin saturation, the proportion of oxygen-saturated to total hemoglobin in the blood, is associated with numerous disorders and is a predictor of health outcomes including mortality, incident heart failure, and dementia. Despite the fundamental role of oxygen saturation in normal and abnormal physiology, there are few large-scale genetic studies of oxygen saturation performed across populations. Overnight measurements provide more variability than daytime levels due to the “stresses” associated with normal and disordered breathing, and also provide an important measure of sleep apnea severity, a common disorder in the population that is associated with considerable morbidity. In this study, for the first time, we identified multiple replicated genome-significant associations based on up to 22,736 individuals from 10 cohort studies. Our findings suggest a contribution of inflammatory genes such as the Interleukin 18 receptor subunit genes to the genetic architecture of sleep-disordered breathing. These results extend our understanding of the genetics of oxyhemoglobin saturation and sleep-disordered breathing and may provide further insight into the biology of associated diseases.

## Introduction

Arterial oxyhemoglobin saturation is a fundamental physiological trait that is tightly regulated at cellular and systemic levels to optimize tissue oxygen delivery. Reduced values, or hypoxemia, occurs secondary to acute and chronic respiratory or cardiovascular diseases, and rarely due to hemoglobin protein mutations. Chronically, low oxygen saturation predicts cognitive deficits in patients with chronic obstructive pulmonary disease (COPD) and in sleep apnea (SA) [1,2].

Oxyhemoglobin saturation (SpO_2_), the proportion of oxygen-saturated to total hemoglobin in the blood, is most commonly measured using non-invasive equipment (oximeters). Oximetry is used to screen and monitor a wide range of health conditions. Normal SpO_2_ values range from 95% to 100% during wakefulness and normally fall by 2 to 4% during sleep. Oxygen saturation is reduced in individuals living at high altitude and in patients with cardiopulmonary diseases. However, even within specific disease groups, there is variation in SpO_2_ that is not explained by factors such as age, obesity, lung function or tobacco exposure [3]. Twin studies indicate that as much as 26% of the variation in waking SpO_2_ can be explained by genetic factors [4]. Population studies also indicate that genetic effects contribute to variation in waking SpO_2_ among Tibetan highlanders and in COPD [3,5].

Sleep disordered breathing (SDB) is a common disorder characterized by recurrent falls of SpO_2_ during sleep due to repetitive episodes of apneas (no airflow) or partial airflow (hypopneas), most often due to recurrent collapse of the upper airway. Our prior family-based studies have indicated that average SpO_2_ during sleep is significantly heritable [6]. Sleep-related hypoxemia is a key component of the pathophysiology of the disorder and variations in SpO_2_ during sleep in patients with SDB are predictive of incident atrial fibrillation [7], certain types of cancer [8–10], and death [7]. Minimum nocturnal SpO_2_ predicted future carotid plaque burden in the Wisconsin sleep cohort, even after adjusting for traditional risk factors [11]. In cohorts of patients with both heart failure and SDB, overnight hypoxemia is a stronger risk factor for incident cardiovascular events and death than is the apnea hypopnea index (AHI, a count of the number of breathing pauses per sleep hour) [12–14]. Mean oxygen saturation and acute hypoxemia during sleep have more significant associations with liver steatosis than the AHI [15]. Sleep-related hypoxemia also may significantly influence prognosis of patients with COPD, asthma, and interstitial lung disease [16–19]. Adverse effects of sleep-related hypoxemia include those directly related to tissue ischemia as well as to the effects related to activation of hypoxia-inducible factor-1 (*HIF1A*) mediated and NF-kB pathways, that then activate the sympathetic nervous system, stimulate release of angiogenic and inflammatory factors, cause oxidative stress, reduce insulin sensitivity, and cause endothelial dysfunction. Therefore, understanding variation in nocturnal SpO_2_ is important for improving our understanding of variation in risk of a wide range of chronic health outcomes.

In this study, we conducted the first multi-ethnic genome-wide association study (GWAS) of 3 nocturnal oxygen hemoglobin saturation (SpO_2_) traits: average and minimum SpO_2_, and the percent of sleep time under 90% SpO_2_ (Per90). (Lower values of Per90 are better while higher values are better for the other two measures.) These measures provide complementary information on hypoxemic burden across the sleep episode and can be derived from oximetry, which is potentially scalable for large-scale studies.

We analyzed data from 10 cohort studies and four ethnic groups and focused on identifying obesity-independent loci by adjusting for body mass index (BMI). We also considered sex differences in associations given the growing interest in sexual dimorphism in genetic analyses [20]. Furthermore, SDB prevalence varies by sex [21,22]), as do SDB risk factors such as the ventilatory response to arousal and regional fat distributions [23,24], and sex differences have also been reported for the relationship between chronic hypoxemia and cardiovascular events [12]. These analyses extend our prior report of results for SDB in Hispanic/Latino-Americans [25].

## Results

### Study sample and cross-phenotype correlations

Descriptive characteristics of the discovery and replication study samples are provided in **Tables 1 and 2**. Collectively, we studied 22,736 individuals. The discovery sample consisted of 8,326 individuals across 6 studies and 4 populations (1,209 African-Americans [AA]; 228 Asian-Americans [AsA]; 5,649 European-Americans [EA]; 1,240 Hispanic/Latino-Americans [HA]). Replication cohorts included 14,410 individuals (681 AAs, 2,378 EAs and European-Australians, and 11,351 HA) from 4 cohorts. Across cohorts, mean age ranged from 37.8 (CFS African-Americans) to 77.7 years (CHS European-Americans). Female participation ranged from 0% (MrOS) to 72% (Starr). The mean BMI ranged from 24.1 kg/m^2^ (MESA Asian-Americans) to 32.3 (JHS). Waking SpO_2_ values, which were typically measured prior to the sleep episode on the same equipment, ranged from 94.96% (in the older MrOS cohort) to 97.78% (among relatively young CFS African-Americans). Average SpO_2_ during sleep ranged from 93.74% (CFS European-Americans) to 96.45% (HCHS/SOL). The median Apnea Hypopnea Index ranged from 1.97 (HCHS/SOL, a relatively young cohort) to 24.60 (WASHS, a sleep clinic-derived cohort). Average forced vital capacity (FVC; percent predicted value) exceeded 90% in cohorts in which these data were available. The prevalence of chronic lung diseases (asthma, COPD) and diabetes varied across cohorts, likely reflecting differences in age, ascertainment and possible disease definitions.

**Table 1:** Discovery sample description.

Ethnic Group	Cohort	N	Age	Percent Female	BMI	Sleep Episode SpO_2_	Minimum Sleep Episode SpO_2_	Percent Sleep Under 90% SpO_2_	Apnea Hypopnea Index	AHI (% <5, 5–15, > = 15)	Waking SpO_2_	FVC (% Predicted)	% Asthma	COPD	% Diabetes
African-American	CFS*	719	37.8 (19.4)	55.6	31.6 (9.6)	94.57 (3.77)	85.82 (9.88)	4.58 (13.14)	5.61 (19.68)	47.1, 21.2, 31.7	97.78 (1.91)	95.10 (19.67)	21.5	15.9	15.8
	MESA	490	69.1 (9.1)	54.3	30.4 (5.7)	94.44 (1.96)	82.93 (8.17)	4.01 (9.46)	13.34 (21.16)	21.2, 32.2, 46.5	96.11 (1.35)	96.02 (17.62)	5.3	14.1	28.0
Asian-American	MESA	228	68.1 (9.2)	50.4	24.1 (3.2)	94.96 (1.22)	83.42 (7.38)	2.17 (4.30)	13.97 (23.90)	22.4, 30.3, 47.4	96.18 (1.01)	98.45 (15.49)	4.4	16.6	16.0
European-American	ARIC	1,432	62.4 (5.7)	51.5	28.8 (5.1)	94.47 (1.99)	85.62 (6.12)	3.36 (10.33)	8.67 (15.55)	3.0, 34.5, 32.5	96.08 (1.70)	102.39 (13.48)	7.3	0.9	6.1
	CFS*	692	41.6 (19.5)	52.8	30.2 (8.7)	93.74 (3.67)	86.46 (9.07)	4.29 (12.30)	5.52 (18.17)	48.6, 20.7, 30.8	96.96 (2.01)	95.68 (18.05)	15.4	18.6	9.2
	FHS*	640	59.4 (9.0)	50.0	28.5 (5.0)	94.68 (1.96)	85.71 (6.07)	2.79 (8.16)	8.20 (14.42)	34.5, 35.5, 30.0	96.15 (1.86)	101.45 (13.41)	7.7	0.4	5.3
	MESA	707	68.5 (9.1)	53.6	28.0 (5.2)	93.93 (1.75)	83.49 (7.41)	4.36 (10.84)	12.62 (20.67)	21.6, 34.4, 44.0	95.70 (1.40)	94.43 (14.08)	2.0	13.6	11.1
	MrOS	2,178	76.7 (5.7)	0.0	27.2 (3.7)	93.85 (1.73)	84.30 (6.08)	4.41 (9.89)	12.74 (18.13)	21.2, 35.0, 43.8	94.96 (1.63)	98.99 (18.33)	7.5	5.3	13.0
Hispanic/ Latino-American	MESA	458	68.3 (9.2)	52.8	30.1 (5.5)	94.33 (1.56)	81.50 (9.38)	3.84 (7.35)	16.94 (23.05)	17.2, 27.9, 54.8	96.12 (1.37)	94.42 (14.91)	5.5	9.2	27.6
	Starr	782	52.3 (11.3)	71.9	32.2 (6.8)	94.65 (2.09)	85.78 (7.50)	2.83 (8.79)	10.35 (17.18)	31.5, 31.5, 37.1	95.93 (2.43)	N/A	N/A	N/A	47.90

Six studies included 8,326 individuals with genotypes and phenotypes (1,209 African-Americans; 228 Asian-Americans; 5,649 European-Americans; 1,240 Hispanic/Latino-Americans). Values are displayed as mean (SD), except for the skewed Apnea Hypopnea Index, which is displayed as median (IQR). Waking O2 saturation values were based on point measurements collected prior to the sleep episode.

*: Family cohort.

**Table 2:** Replication sample description.

Ethnic Group	Cohort	N	Age	Percent Female	BMI	Sleep Episode SpO_2_	Minimum Sleep Episode SpO_2_	Percent Sleep Under 90% SpO_2_	Apnea Hypopnea Index	AHI (% <5, 5–15, > = 15)	Waking SpO_2_	FVC (% Predicted)	% Asthma	% COPD	% Diabetes
African-American	CHS	185	75.7 (4.8)	59.5	28.7 (4.8)	95.01 (2.07)	85.69 (5.34)	3.16 (8.84)	11.42 (16.67)	25.4, 36.8, 37.8	96.16 (1.99)	96.30 (23.21)	11.29	3.33	22.58
	JHS	496	62.7 (10.8)	63.1	32.3 (7.0)	94.72 (2.06)	84.07 (6.52)	3.20 (9.19)	10.87 (14.65)	24.7, 39.6, 35.7	N/A	N/A	8.23	4.32	22.43
European-American	CHS	731	77.7 (4.2)	60.6	27.3 (4.4)	94.13 (1.91)	84.77 (6.39)	4.24 (11.32)	10.82 (15.47)	48.6, 20.7, 30.8	95.45 (1.87)	90.69 (18.80)	6.72	1.81	10.25
European-Australian	WASHS	1,647	52.1 (13.7)	40.2	32.0 (7.8)	N/A	83.95 (9.39)	6.11 (15.10)	24.60 (30.80)	4.7, 24.5, 70.9	95.13 (2.48)	91.68 (14.59)	25.8	17.0	13.96
Hispanic/ Latino-American	HCHS/SOL	11,351	46.2 (13.8)	59.1	29.8 (6.0)	96.45 (0.95)	87.07 (6.05)	0.85 (3.14)	1.97 (6.20)	68.9, 19.4, 11.7	96.94 (3.13)	94.34 (15.71)	7.70	2.78	19.55

Four studies included 14,410 individuals with genotypes and phenotypes (681 African-Americans; 2,378 European-Americans and European-Australians; 11,351 Hispanic/Latino-Americans). Values are displayed as mean (SD), except for the skewed Apnea Hypopnea Index, which is displayed as median (IQR). Waking O2 saturation values were based on point measurements collected prior to the sleep episode.

Pairwise correlations among phenotypes and selective demographic variables are shown in **S1 Table**. As expected, the three overnight oxyhemoglobin saturation traits are strongly correlated (average SpO_2_ –minimal SpO_2_ ρ = 0.61; average SpO_2_ –Per90 ρ = -0.73; minimal SpO_2_ –Per90 ρ = -0.86). Waking oxyhemoglobin saturation correlates with average nocturnal SpO_2_ (ρ = 0.59), minimal nocturnal SpO_2_ (ρ = 0.35) and Per90 (ρ = -0.40). The AHI was also correlated with minimal SpO_2_ (ρ = -0.71), Per90 (ρ = 0.70), and average SpO_2_ (ρ = -0.55). Lung function (percent predicted FEV_1_ and FVC [26]) correlated modestly with each of the overnight oxygen saturation measures (ρ = -0.20 –+0.23).

### Meta-analysis results overview

**Fig 1:**
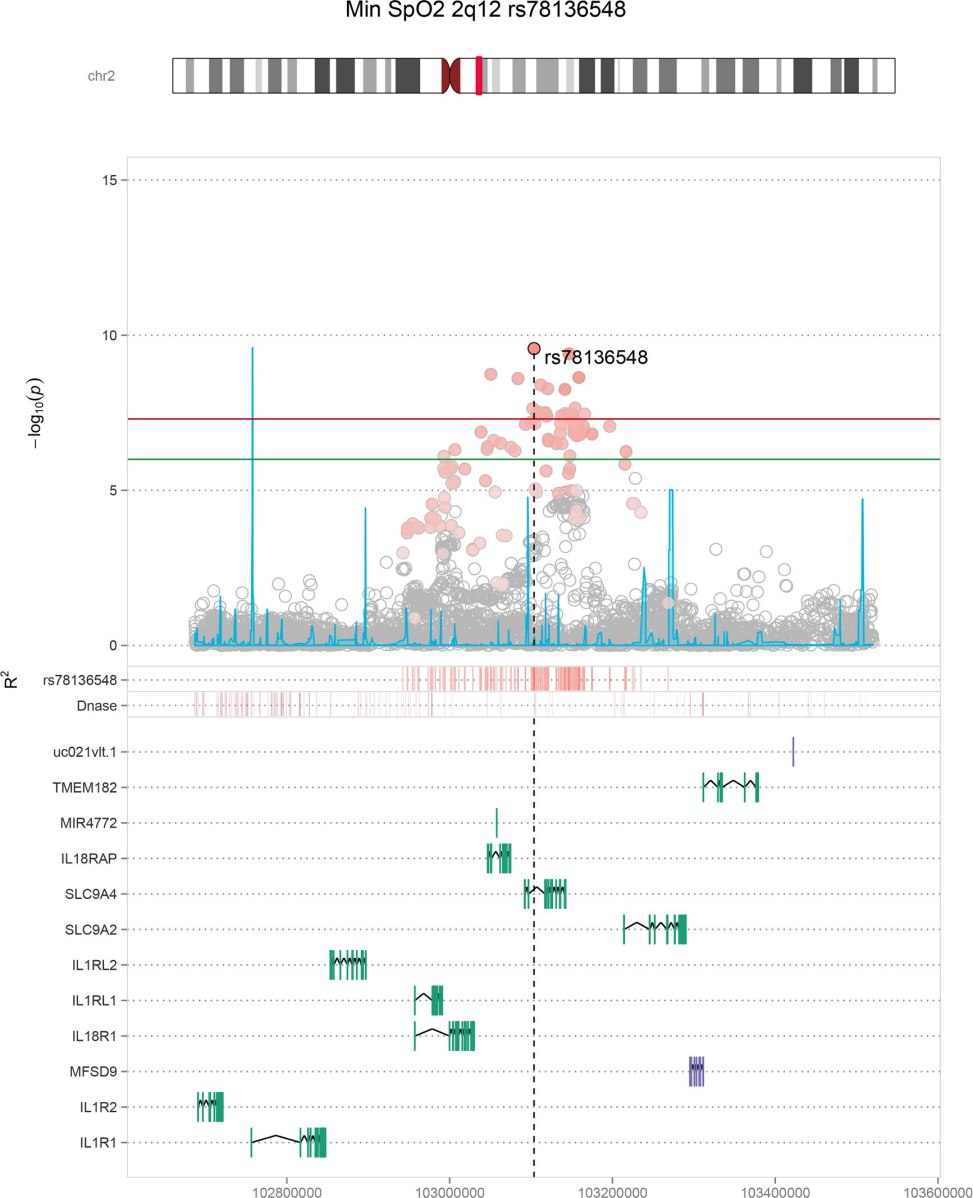
Minimum oxygen saturation 2q12 regional association plot. Physical positions (Build 37 coordinates) are shown on the X-axis. The main graphic shows log-transformed p-values for individual SNPs on the Y-axis. SNP colors indicate the degree of linkage disequilibrium (LD) with the lead SNP rs78136548 (based on combined 1000 Genomes AFR, AMR, and EUR populations). The significance cut-off of p = 5 × 10^−8^ is shown with a horizontal red line. The blue line denotes recombination rates. Lower tracks indicate positions of SNPs with strong LD with rs78136548, regions of Dnase hypersensitivity sites, and exon positions for local genes.

**Fig 2:**
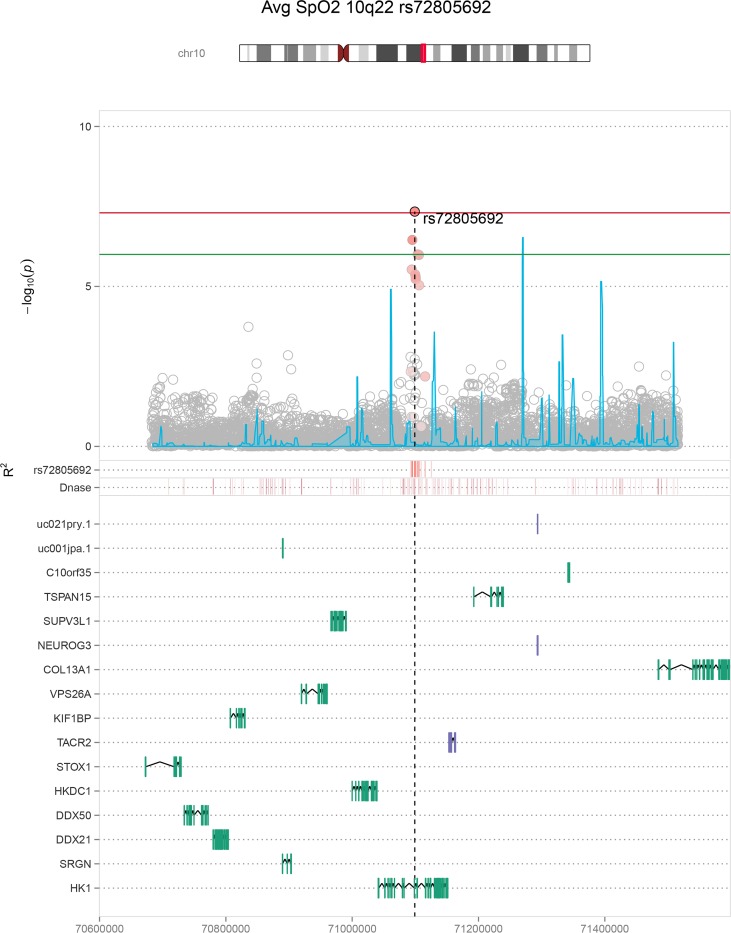
Average oxygen saturation 10q22 regional association plot. This figure depicts the multi-ethnic average oxygen saturation results in the *HK1* (hexokinase 1) region, which had the highest sample size among all significant *HK1* region associations (n = 20,676). Other significant and suggestive *HK1* regional associations are shown in S7, S11 and S17 Figs.

Manhattan and QQ plots for the overall sample and population-specific primary discovery analyses are provided in **S1–S3 Figs**. The maximum lambda value was 1.02, in the multi-ethnic average SpO_2_ analysis, suggesting that our analysis results were largely free of technical artifacts and corrected appropriately for population structure within each ethnic group. We analyzed SNPs in loci with discovery p-values < 1 × 10^−6^ in our replication cohorts and identified 6 significant (p < 5.0 × 10^−8^) and 1 suggestive (p < 1.0 × 10^−6^) associations in joint discovery and replication analyses spanning 5 regions; 2q12 (*IL18R1*; **Fig 1**), 10q22 (*HK1*; **Fig 2**), 3p24 (intergenic region; **S8 Fig**), 4q35 (RP11-242J7.1, **S9 Fig**); **S2 Table**), with several associations specific to given population backgrounds. Effect estimates and directions of allelic effect were consistent in the discovery and replication stages for all SNPs (METAL heterogeneity p > 0.1).

### Interleukin 18 Receptor 1 and Hexokinase 1 region meta-analysis results

In the multi-ethnic combined discovery and replication meta-analysis (n>20,000), genome-wide significant associations were identified with: a) minimum SpO_2_ in the *IL18R1* region (rs78136548 discovery, p = 2.66 × 10^−7^, combined p = 2.70 × 10^−10^); and b) average SpO_2_ in the *HK1* region (rs72805692 discovery p = 7.20 × 10^−8^, combined p = 4.58 × 10^−8^) (**Figs 1 and 2, Table 3**). Lead population- and sex-specific SNPs from each locus meeting our replication criteria definitions are also presented **(S2 Table)**. Consistent negative effect directionality was observed for the *IL18R1* region SNP rs78136548 T across all 13 available association tests in African, European, and Hispanic/Latino ancestral populations (**S3 Table**). The association was largely driven by males (females beta(se) -0.065 (0.023), p = 0.005, males beta(se) -0.131 (0.022), p = 2.69 × 10^−9^; **S4 Table**). Average SpO_2_ was significantly associated with the *HK1* region in a European-American analysis (rs16926246 n = 5,649; p = 2.46 × 10^−8^; 1000G EUR rs72805692 r^2^ = 0.625). The *HK1* region was also notable for a second European-American significant association using the complementary phenotype Per90 (percentage of sleep with oxyhemoglobin saturation below 90%; rs148471505 p = 3.08 × 10^−8^; 1000G EUR rs72805692 r^2^ = 0.679; **S2 Table**). Minimum SpO_2_ was also suggestively associated with the *HK1* region in European-American males (rs17476364 p = 6.79 × 10^−8^; 1000G EUR rs72805692 r^2^ = 0.937; **S3 Table**).

**Table 3:** Significant *IL18R1* and *HK1* region meta-analysis results.

Region	Phenotype	Model	SNP	Suggestive Regional SNPs	Combined N	CAF	Discovery Beta (SE)	Discovery P	Replication Beta (SE)	Replication P	Combined Beta (SE)	Combined P
*IL18R1*	Min SpO_2_	All	rs78136548 T	66	22,333	0.866–0.953	-0.124 (0.024)	2.66 × 10^−7^	-0.082 (0.021)	1.01 × 10^−4^	-0.101 (0.016)	2.70 × 10^−10^
*HK1*	Avg SpO_2_	All	rs72805692 G	2	20,676	0.017–0.112	0.141 (0.026)	7.20 × 10^−8^	0.060 (0.025)	1.46 × 10^−2^	0.098 (0.018)	4.58 × 10^−8^

Lead significant (p < 5.0 × 10^−8^) *IL18R1* (2q12) and *HK1* (10q22) region SNP multi-ethnic (n > 20,000) meta-analysis associations are shown. Suggestive regional SNPs denotes the count of SNPs with p < 1.0 × 10^−6^. CAF indicates coded allele frequency range. SNP columns include the coded allelle. Individual regional SNP results are provided in S3 Table, with sex-stratified analyses of each locus SNP provided in S4 Table. Stage-specific analyses (lead loci SNPs only) are provided in S5 Table.

Given that sleep disordered breathing and respiratory control vary by sleep state [27,28], we also explored whether associations for oxygen saturation differed when using measurements specific to non rapid eye movement (NREM) versus rapid eye movement (REM) sleep in cohorts with sleep state information (**S5 Table**). Several SNPs showed associations with lower p-values and/or higher point estimates for stage-specific results. The minimum SpO_2_ within NREM rs72805692 association result showed the lowest p-value for any *HK1* locus SNP across all analyses (p = 1.60 × 10^−9^) and further indicated that the *HK1* region SNPs were significantly associated with all three traits.

### Additional analyses

The secondary sex-stratified discovery analyses (Miami plots, **S4–S6 Figs)** identified 6 additional independent loci associated in males with consistent effects in replication and suggestive p-values < 1.0 × 10^−6^ in joint analyses (**S3 Table; S11–S17 Figs**). A combined secondary meta-analysis of the discovery and replication cohorts for SNPs with initial discovery phase p-values ≥ 1.0 × 10^−6^ identified four additional significantly associated loci (22q11, 6q25, 17p13 and 7q22 including new candidate genes *CHRNE* and *RELN*; **S6 Table** and **S18–S22 Figs**). Joint analysis results of the lead loci are provided for each SNP in **S3 Table**, while a comparison of individual SNP results using combined-sex and sex-stratified models can be found in **S4 Table**.

### GWAS results overlap across oxygen saturation traits, apnea hypopnea index, and pulmonary traits

Although overnight oxygen saturation values most strongly correlate with measurements of SDB, they also may be influenced by pulmonary function, waking oxygen saturation, and hemoglobin levels. We therefore tested associations of the lead loci for sleep SpO_2_ traits with waking SpO_2_, the AHI (the clinical metric for SDB), forced vital capacity (FVC, percent predicted, a measure of pulmonary function), and hemoglobin concentration (**S5 Table**). Sample sizes for the comparison traits varied based on the availability of these exploratory phenotypes, with hemoglobin and FVC collected at different exams from the sleep recordings in a subset of cohorts. Consistent with the strong correlation between sleep period SpO_2_ and AHI phenotypes (S1 Table), several lead oxygen saturation SNPs also displayed modest to strong associations with the AHI (12 of 17 available p-values < 0.05; minimum AHI p = 5.3 × 10^−5^). Weaker associations were observed with the other traits: waking SpO_2_ (p generally > 0.01 and < 0.05; minimum p = 0.005); FVC (p generally > 0.05; minimum p = 0.025); and hemoglobin concentration (p generally > 0.05; minimum p = 0.003). We next evaluated whether associations between sleep SpO_2_ values and lead SNPs persisted after adjusting for AHI, FVC percent predicted, waking SpO_2_, asthma history, COPD history, current smoking status, and hemoglobin concentration (**S8 Table**). Analyses restricted to individuals with these available covariates showed that neither FVC, asthma, COPD, current smoker status or hemoglobin concentration changed the estimated SNP effects for sleep SpO_2_ by more than 10% for any SNP, suggesting that lung function and lung disease did not mediate the associations between sleep oxygen saturation and each SNP. In contrast, adjustment for AHI reduced the SNP association effect estimates by more than 10% in most of the models tested. Adjustment for waking SpO_2,_ which correlated with nocturnal oxygen saturation_,_ reduced the effect estimates by 0.2 to 35% (S8 Table), although the statistical significance of direct associations of these SNPs with waking SpO_2_ was modest (p = 0.003–0.93; S5 Table).

### Correlated functional and eQTL SNPs and cell line enhancer enrichment: Bioinformatics data

We searched for SNPs in our top loci that overlap regulatory regions as determined by the ENCODE and Roadmap Epigenomics Consortia and collated by HaploReg. **S9 Table** lists the 144 of 227 unique SNPs with p-values < 1 × 10^−6^ that overlap regulatory regions (promoter or enhancer marks; DNase I hypersensitivity sites; or protein binding regions) in at least 1 cell line. Our lead *HK1* region SNP in multi-ethnic meta-analysis, rs72805692 (average SpO_2_ multi-ethnic p = 4.58 × 10^−8^) overlapped enhancer marks in 107 cell lines across 21 organs. Other notable genome-level significant SNPs in the *HK1* locus include rs16926246 and rs148471505 (overlapping enhancer marks in 91 and 71 cell lines, respectively). We further queried for Blueprint Consortium promoters and enhancers (largely blood cell lines) and Vermunt *et al*. brain region enhancers (**S10 Table**). 104 of the 227 unique replication and combined meta-analysis SNPs with p-values < 1 × 10^−6^ overlapped at least one regulatory region. rs72805692 additionally overlapped 30 Blueprint and 98 Vermunt enhancer regions, and rs16926246 overlapped 106 combined enhancer regions.

We also queried overlap with published expression quantitative trait loci (eQTL) associations. 182 of the 227 unique SNPs with p-values < 1 × 10^−6^ were eQTL SNPs for at least one of 42 genes (**S11 Table**). 13 SNPs that were genome-level significant SNPs in the *IL18R1* region were also eQTL SNPs for both interleukin 18 receptor subunits in whole blood (*IL18R1* eQTL p < 4.9 × 10^−11^; *IL18RAP* eQTL p < 1.6 × 10^−46^), indicating a possible role for interleukin 18 signaling in the as yet unknown causal tissue(s). No significant colocalization was observed when testing this region using Blueprint Consortium eQTL signals. The lead significant *HK1* region SNP rs16926246 was also associated with *HK1* expression in whole blood (EA average SpO_2_ p = 2.46 × 10^−8^; *HK1* eQTL p = 9.64 × 10^−13^).

### Gene and pathway analyses

We performed a multi-variate GWAS of the three SpO_2_ traits in the European-ancestry samples using MTAG [29]. Lead results (p < 1 × 10^−6^) are shown in **S12 Table**. No novel genome-level significant loci were detected.

We used our European-American GWAS meta-analysis results to impute gene-level expression differences in a subset of 6 GTEx-assayed tissues and Depression and Genes and Networks (DGN) whole blood using MetaXcan. Tissue-specific results are presented in **S13–S19 Tables**. Three genes were associated at either a Bonferroni-adjusted significance level (p < 4.01 × 10^−7^) or at a suggestive level within an order of magnitude, all in the minimum SpO_2_ analysis: *CHRNE* (minimum p = 7.61 × 10^−8^ in subcutaneous adipose tissue), *C17orf107* (overlapping and antisense to *CHRNE*; minimum p = 2.68 × 10^−7^ in visceral omentum adipose tissue), and *IL18R1* (minimum p = 6.28 × 10^−7^ in subcutaneous adipose tissue).

We carried the whole blood MetaXcan results forward for pathway analyses (DGN sample size = 922). GIGSEA analyses of KEGG pathways and Molecular Signatures Database curated microRNAs and transcription factors are presented in **S20–S22 Tables** respectively. The most enriched KEGG pathway was steroid hormone biosynthesis (Average SpO2 empirical p-value = “0” following 10,000 permutations). This pathway was observed twice with empirical p-values < 0.05, as were the KEGG asthma and ribosome pathways. The most significantly observed miRNA binding site was for MIR-380-3P (average SpO2 empirical p-value = 0.006). MIR-140 and MIR-190 displayed empirical p-values < 0.05 in two analyses. *PPARG* transcription factor binding sites were enriched in all three analyses, while PPAR signaling was the most enriched Per90 KEGG pathway. *NHLH1* (formerly *HEN1*) transcription factor binding sites were enriched in all three analyses, and six transcription factor binding sites were enriched in two analyses.

## Discussion

Novel associations were identified for several genetic loci with traits measuring oxyhemoglobin saturation during sleep in a large, multi-ethnic population. The traits examined quantify overnight hypoxemia, a key component of sleep disordered breathing that predicts risk of developing cognitive impairment, cardiovascular disease, atrial fibrillation, and mortality in community and clinical cohorts [2,7,12–14]. Although nocturnal hemoglobin oxygen saturation level inversely correlates with the number of breathing pauses at night (apneas, hypopneas), there is much individual variation in sleep-associated hypoxemia that is not well understood. For the first time, this study identified genetic variants associated with oxyhemoglobin saturation traits measured during sleep. Specifically, we identified and replicated variants in two gene regions- hexokinase 1 (*HK1*) and interleukin 18 receptor (*IL18R1*)- that individually and together are of potential high relevance to lung and ventilatory-control pathobiology. In addition, we identified several other loci of potential biological significance.

### HK1 and IL18R1 regional associations

We identified significant associations between *HK1* SNPs and average oxygen saturation (SpO_2_) during sleep and percentage of sleep with SpO_2_ < 90% (Table 3). State-specific results also indicate a genome-level association with minimum SpO_2_ during NREM sleep (S5 Table). Hexokinase is the first enzyme and the rate-limiting step in the glycolysis pathway [30,31] and its activity is regulated by hypoxia inducible factor 1a (*HIF1A*) [5,32,33]. Obstructive sleep apnea following CPAP withdrawal increases glucose during sleep [34]. Rs16926246 (significantly associated with average SpO_2_) and rs10159477 (among our suggestive average SpO_2_ SNPs, S3 Table) are associated with *HK1* expression in whole blood ([35], S11 Table) and also have been associated with hemoglobin concentration [36]. Rs16926246 was the study-wide lead SNP and previously found to be highly significantly associated with hemoglobin A1c (HbA1c) levels, a marker of glucose homeostasis, through an erythrocytic pathway [37]. Rs72805692, our lead multi-ethnic average SpO_2_ SNP, has also been associated with HbA1c levels [38]. Both SNPs overlap enhancer marks in ≥ 197 ENCODE, Roadmap Epigenomics, Blueprint, and Vermunt *et al*. cell lines and/or brain regions (including erythroblasts; S9 and S10 Tables). The Rapoport–Luebering glycolytic shunt affects erythrocytic oxygen capacity through allosteric binding of 2,3-bisphosphoglycerate (2,3-BPG, also known as 2,3-diphosphoglycerate or 2,3-DPG) to hemoglobin. The concentration of glycolytic pathway intermediates can impact 2,3-BPG concentration, partially mediated by hexokinase [39–41]. Rs72805692 was marginally associated with hemoglobin concentration in our sample for individuals with available measurements (p = 0.0034). The A allele was associated with reduced hemoglobin concentration, average sleeping and waking SpO_2_ and increased sleep time with oxygen saturation under 90%. However, the association with average sleeping SpO_2_ was not appreciably changed when adjusting for hemoglobin concentration (S5 Table).

Alternatively, *HK1*, in concert with cytokines including *IL18*, may influence overnight oxygen saturation through effects on pulmonary inflammation and ventilation-perfusion mismatch. Hexokinase-1 mediated glycolysis regulates the NLR Family Pyrin Domain Containing 3 (*NLRP3*) inflammasome [42], a multiprotein complex implicated in obesity-related inflammation [43] as well as several pulmonary diseases [44–50]. The *NLRP3* inflammasome activates caspase-1, resulting in cleavage of pro-IL1B and pro-IL18 into their mature forms, amplifying inflammation [50,51]. The *NLRP3* inflammasome is proposed to play a critical role in lung injury occurring in response to exposures to inflammatory mediators, oxidative stress and mechanical ventilation, including cyclic pulmonary stretching, which induces *NLRP3* inflammasome activation in alveolar macrophages [52]. Patients with SDB, particularly obstructive sleep apnea, experience oxidative stress and pulmonary inflammation [53], as well as swings in intrathoracic pressure, potentially causing pulmonary strain. Our data suggest the possibility that variations in *HK1* (and possibly *IL18*) pathways may contribute to individual differences in pulmonary gas exchange occurring during sleep, possibly through pulmonary inflammation and/or subclinical pulmonary injury. Circulating markers of alveolar epithelial injury, including KL-6, surfactant protein-A, and matrix metalloproteinase-7, correlate with degree of overnight hypoxemia and AHI in patients with SDB [54,55]. Chronic intermittent hypoxia induces physiological deficits in rats with allergen-induced airway inflammation, due to collagen deposition and other effects [56]. Although the mechanisms underlying these associations are unclear, lung imaging studies show an increase in subclinical interstitial lung abnormalities in individuals with sleep apnea [55]. Our data suggest the possibility that variations in *HK1* pathways may contribute to differences in pulmonary gas exchange that occurs during sleep, possibly through effects on ventilation-perfusion mismatch due to subclinical pulmonary inflammation. Recent population-based studies found that sleep apnea associates with both elevated pulmonary inflammatory markers and imaging evidence of interstitial lung abnormalities [55]. Finally, the *NLRP3* inflammasome has been shown to influence brain tissue, and changes in sleep delta power have been observed in knock-out mice [57].

Another and correlated mechanism could be through *HIF1A*. In addition to oxygen sensing effects in the carotid body [58], HIF1A regulates HK1 in human alveolar cells [59], is regulated by PFKM (a downstream glycolysis enzyme) in macrophages [60], and is involved with metabolic reprogramming of macrophages [61]. Activation of glycolytic enzymes in pulmonary epithelial cells exposed to cyclic mechanical stretching is abrogated with *HIF1A* repression [62]. Ventilatory differences in responses to intermittent hypoxemia secondary to SDB could influence several measures of overnight SpO_2_, as observed in our analyses.

The second set of significant SNPs implicated in overnight SpO_2_ levels were in the *IL18R1* region, with external evidence indicating eQTL associations with both *IL18* receptor subunit genes (*IL18R*, *IL18RAP*) in over a dozen genome-level significant SNPs (S8 Table). Minor alleles were associated with an increase in minimum oxygen saturation, an increase in *IL18RAP* expression, and a decrease in *IL18R1* expression in whole blood [35]. *IL18R1* expression was also suggestively associated with minimum oxygen saturation in a gene-level analysis using MetaXcan. These genes are essential for *IL18* signaling [63,64], suggesting that the *IL18* pathway may partially mediate the association at this locus. *IL18* is a pro-inflammatory cytokine produced by macrophages and is involved in multiple inflammatory disorders [65]. This region has been associated with Blautia genus microbiota abundance in the gut (lead locus SNP rs79387448 Min SpO2 p = 8.61 x 10–8 [S3 Table]) [66]. *Il18* over-expression in mice leads to chronic pulmonary inflammation, including the increased levels of CD4+, CD8+ CD19+, eosinophils, macrophages, NK1.1+, and neutrophils; along with alveolar destruction, fibrosis, and other effects [67,68]. In humans, IL18 plasma concentration levels are elevated in acute respiratory distress syndrome [69]. As described above, IL18 as well as IL1B are the inflammatory proteins activated by caspase 1 in *HK1*-regulated *NLRP3* inflammasome activation [42]. MTOR, a member of the complex that induces this HK1-mediated activation, is the 6^th^ most associated gene in our analyses of Per90 using MetaXcan whole blood analysis (p = 4.5 × 10^−4^, S18 Table). Mechanical stretching-induced NLRP3 inflammasome activation induces activated IL18 release from alveolar macrophages [52]. *Casp1*- (required for Il18 activation) and *Nlrp3*-knockout mice are protected from hypoxemia accompanying mechanical ventilation [70]. Models of cyclic stretching induce the release of Il18 in mouse alveolar macrophages, mediated by *Nlrp3* [52]. Serum IL18 concentrations are also significantly higher in patients with SDB compared to obese controls and correlated with serum concentrations of C reactive protein and interleukin 6 [71]. *Il18r1* is among the genes with the most robust circadian rhythmic profiles in healthy mouse lung [72]; it is possible that timing-specific gene expression may influence the nocturnal hypoxemia phenotype we studied. An association between asthma and a SNP in the *IL18R1* region is reported [73]. Our lead SNPs had reduced linkage disequilibrium with this SNP (rs3771166 minimum p in any model = 5.6 x 10^−5^ for minimum SpO_2_ in EA males). Analyses adjusted for asthma did not significantly attenuate our associations (rs78136548 minimum SpO_2_ p adjusted for asthma = 1.21 x 10^−7^, unadjusted p = 1.01 x 10^−7^ in equivalent samples). Therefore, different variants in the *IL18R1* region may influence pulmonary and sleep related hypoxemia traits. The circadian timing of sleep may impact *IL18* pathway-specific effects on pulmonary-related traits. The associations with *HK1* further also implicate the possibility that variants in both genes may contribute to SpO_2_ levels. *IL18* has also been shown to regulate *HIF1A* [74].

### Other regional associations

The protein coding gene most proximal to the Per90 association with the 4q35 region in African-Americans (S2 Table, S9 Fig) was *CASP3*, a second caspase gene involved with alveolar wall destruction [75]. Despite the modest sample size, the p-value almost met genome-wide significance (p = 9.39 x 10^−7^), suggesting the utility of future studies of the role of this gene in influencing nocturnal saturation.

We also detected multiple genome-level significant associations following a combined discovery plus replication cohort meta-analysis (S7 Table). Although these associations require independent evidence for replication, two of these associations are of particular interest. In European-Americans, minimum oxygen saturation was associated with 2 genome-level significant and 65 suggestive SNPs that are associated with *CHRNE* (acetylcholine receptor, nicotinic epsilon [muscle]) expression in 19 GTEx tissues and monocytes (p < 5 x 10^−8^; S11 Table). *CHRNE* and the proximal gene *C17orf107* had the lowest p-values in the expression-based MetaXcan gene tests. Phospholipidase D2 (*PLD2*), another gene associated with the locus through expression (eQTL) SNPs, is required for hypoxia-induced expression of *HIF1A*. Hypoxia-induced gene expression in mouse lung endothelial cells is reduced in *Pld2* knockout mice [76]. A multi-ethnic Per90 association physically overlaps *RELN* (reelin). While no expression or epigenetic evidence was available, this association is of interest given the suggested respiratory role of reelin within the Pre-Bötzinger complex, a major center for respiratory control [77].

No novel genome-level significant loci were detected in our multi-variate MTAG analyses (S12 Table). As expected from the univariate results, *HK1* was significantly associated and the lead SNP rs72805692 had reduced p-values for average and minimum SpO_2_ (p = 4.0 × 10^−9^ and 1.1 × 10^−9^ respectively). Among the genes in novel suggestive regions was *WLS* (formerly *GPR177*; rs17481104 minimum SpO_2_ p = 5.8 × 10^−7^), which is involved in pulmonary vascular development and has been suggestively associated with airflow obstruction in COPD [78,79]. Enrichment of the KEGG asthma pathway for both minimum SpO2 and Per90 (S20 Table) lends support to the ‘overlap syndrome’ of these two pulmonary diseases [80]. PPAR-gamma transcription factor binding site enrichment in all three analyses (S22 Table) suggests the potential importance of future mechanistic studies of this inflammation-related transcription factor. PPAR signaling was the most enriched KEGG pathway in the Per90 analysis (S20 Table). PPAR signaling and *PPARG* expression in visceral adipose tissue have previously been associated with obstructive sleep apnea [81].

### Sex- and state-specific effects

Sex-stratified analyses identified stronger associations among males compared to females for the *IL18R1* signal (rs78136548 p females = 0.005, males = 2.69 × 10^−9^; S4 Table) and for several SNPs within the *HK1* locus (*e*.*g*. rs17476364 EA min SpO_2_ p females = 0.19, males = 6.79 × 10^−8^). In addition, sex-stratified analyses identified two other loci of interest (S6 Table). Among EA males only, a suggestive association with Per90 in the *IL1RAPL1* region of the X chromosome was identified. The region has recently been suggestively associated with asthma in Hispanic/Latino children, with one replication cohort indicating potential male-specific effects [82]. An association in the *PPP4R1* region (protein phosphatase 4 regulatory subunit 1) was nearly genome-level significant in EA males (p = 5.4 × 10^−8^). Ten suggestive SNPs in this locus were also *PPP4R1* eQTL SNPs in whole blood (p < 1 × 10^−40^; S11 Table). *PPP4R1* regulates *HDAC3* (histone deacetylase 3), an epigenetic modulator of circadian lipid metabolism [83,84].

Respiratory control and neuromuscular activation vary between NREM and REM sleep. Analyses restricting to these states may reduce heterogeneity due to differences in state that influence airway patency or respiratory chemosensitivity. The *PPP4R1* locus p-value lowered to genome-level significance when analyzed using NREM sleep data (rs78805840 p = 1.81 × 10^−8^; S5 Table). The lowest overall p-value in the *HK1* locus was a minimum SpO_2_ within NREM association with rs27805692 (all population combined-sex p = 1.60 × 10^−9^).

### Pulmonary trait effects

The three traits that we analyzed (average and minimum SpO_2_ during sleep, percent of sleep with SpO_2_ < 90%) each are commonly measured and reported in evaluation of patients with sleep apnea. Although correlated, they each measure somewhat different aspects of oxygen saturation. Notably, associations for the Hexokinase 1 (*HK1*) region showed associations with several measures of oxygen saturation. Consistency of these findings across phenotypes suggests the importance of the *HK1* pathway in influencing several aspects of oxygenation during sleep, including severity of response to an airway occlusion (*i*.*e*., as measured by minimal saturation), overall severity (Per90), and overall level (average). Although overnight hypoxemia can occur with underlying pulmonary disease, our analyses also showed that each SpO_2_ association typically associated with the AHI, a primary index of sleep apnea, and associations were not appreciably influenced after considering effects of lung disease, tobacco use, lung function, and hemoglobin level (S8 Table). As expected, baseline oxygen saturation also correlated with average SpO_2_, consistent with an influence of waking SpO_2_ on overall nocturnal levels. Notably, none of our sleep-related trait associations overlapped published associations for resting oxygen saturation in COPD.

Across our cohorts, average level of lung function was within the normal range, and prevalence of lung diseases was low. These findings, as well as our analyses that adjusted for several factors and independently assessed genetic signals for the other pulmonary traits, indicate that the variations in SpO_2_ traits in our cohorts predominantly reflected differences in SDB-related levels of hypoxemia. Relevance of our results to SDB is supported by finding that 12 of the 17 available lead loci SNPs across all analyses had at least a nominal association with the AHI (S5 Table), including the associations for *HK1* rs72805692 and *IL18R1* region rs78136568 SNPs (p = 8.1 × 10^−5^ and 7.2 × 10^−5^ respectively). SDB is a common disorder affecting 17% of middle-aged men and 9% of middle-aged women and characterized by repetitive episodes of upper airway obstruction resulting in intermittent hypoxemia, sleep disruption, and profound physiological disturbances [85]. Past genetic analyses of sleep apnea have mainly focused on the AHI, which does not fully describe the broad range of physiologic stressors that occur in SA, including patterns of SpO_2_ desaturations [86]. Overnight SpO_2_ analysis provides clinically relevant information [2,7–9,12–14] and can be measured relatively simply, and thus can be scaled for future genetic studies and precision medicine.

### Strengths and weaknesses

Our study has several strengths. The sample size of over 22,000 is among the largest available GWAS analyses of any trait associated with objectively recorded sleep disordered breathing. The associations were observed in cohorts with varying demographics and ascertainment strategies (Tables 1 and 2), and as such are likely generalizable to diverse populations. We used a stringent imputation quality threshold (0.88) to reduce random error, using a 1000 Genomes Project or denser template in all studies. Several of our associations are supported by published gene expression, bioinformatics evidence, and/or physiological studies.

Several weaknesses also need to be acknowledged. While we have not performed functional assays as part of the present analysis, the most biologically compelling candidates are supported by several lines of evidence from the literature and will require future experimental validation. Data on potential mediators (*e*.*g*., lung function, hemoglobin) were collected in different visits or were available only in a subset of our cohorts. Some promising findings did not meet genome-wide significance criteria or could not be replicated across cohorts. While this first multi-ethnic meta-analysis of the three traits included over 22,000 individuals, weak or population-specific associations were likely to be missed due to power limitations.

In conclusion, we have performed the first genome-wide association analysis of clinically relevant sleep disordered breathing traits, specifically measures of nocturnal oxygen saturation, and identified several novel associations that are of potential biological relevance. Of particular interest were variants in the *HK1* and *IL18R1* regions. Understanding the genetic underpinnings of these sleep-related traits may guide future studies investigating the contribution of sleep disordered breathing to the hypoxemic burden of pulmonary disorders, and identify common mechanisms such as activation of the *NLRP3*-inflammasome pathway.

Full meta-analysis results are freely available from http://www.sleepdisordergenetics.org/informational/data.

## Material and methods

This research was approved by the Partners Healthcare IRB committee (protocol # 2010P001765). Participant consent was obtained through written documents.

### Discovery group studies

The Atherosclerosis Risk in Communities Study (ARIC; n = 1,432) and Framingham Heart Study (FHS; n = 640) cohorts participating in the Sleep Heart Health Study (SHHS) were analyzed with available polysomnography (PSG) and genotype data [87–89]. This community-based study included a baseline examination (1995–1998) that included in-home polysomnography, and questionnaires [89]. Polysomnography from the baseline examination was collected using the Compumedics PS-2 system (Abbotsford, AU) [90]. Oxyhemoglobin saturation was measured with finger pulse oximetry over the sleep episode and cleaned of signal artifact. The 3 parent cohorts are described below, with CHS used as a replication cohort in the current study (genetic data from this cohort was obtained after the other two studies). In ARIC, genotyping was performed using the Affymetrix 6.0 array. In FHS, genotyping was performed using the Affymetrix 500k and Illumina Omni 5M arrays (obtained from dbGaP; pht000395.v7.p8).

The Cleveland Family Study (CFS) is examining the genetic and familial basis of sleep apnea with 2,534 African- and European-American individuals from 356 families. Four visits occurred from 1990–2006, with a final visit at a clinical research center (2000–2006). Index probands with confirmed sleep apnea were recruited from sleep centers in northern Ohio. Additional family members and neighborhood control families were also studied [91]. Measurements including sleep apnea monitoring, anthropometry, other related phenotypes, and questionnaires. Before 2000, an Edentrace Type 3 home sleep apnea device was used (Eden Prairie, MN). The final examination used 14-channel polysomnography (Compumedics E series, Abottsford, AU). Genotyping was based on the Affymetrix 6.0 and Illumina OmniExpress, Exome, and IBC chip arrays. Data were based on 1,411 individuals with both genotypes and sleep data from either the home sleep study (n = 784) or the lab-based study (n = 627).

The Multi-Ethnic Study of Atherosclerosis (MESA) is examining the risk factors of clinical cardiovascular disease [92]. The baseline examination in 2000 included 6,814 participants ages 45 to 84 from 6 communities: Baltimore MD, Chicago IL, Los Angeles CA, New York NY, Minneapolis/St. Paul MN, and Winston-Salem NC. Four ethnicities are being studied: African-, Asian-, European-, and Hispanic/Latino-Americans. An ancillary sleep study of 2,060 individuals who did not use nightly CPAP, overnight oxygen, or an oral device for sleep apnea occurred between 2010–2013. Sleep measurements included in-home PSG, actigraphy, and a questionnaire adapted from the SHHS questionnaire [93]. Unattended polysomnography used a 15-channel monitor (Compumedics Somte System, Abbotsford, AU). Final study inclusion for individuals with an Affymetrix 6.0 assay was 1,883.

The Osteoporotic Fractures in Men Study (MrOS) is a prospective cohort study examining the risk factors for fractures, osteoporosis, and prostate cancer [94,95] in males age 65 or older from six U.S. communities. An ancillary sleep study of 3,135 individuals was conducted between 2003 and 2005, including in-home PSG (Compumedics Safiro system; Abbotsford AU), anthropometry, and questionnaires. Genotyping was performed with the Illumina Human Omni 1 Quad v1-0 H array. A total of 2,178 European ancestry individuals had PSG and genotype data.

The Starr County Health Studies (Starr) have been examining the risk factors of diabetes in a predominantly Mexican-American border county in Texas since 1981 [96,97]. The sleep apnea assessment occurred between 2010 and 2014 and included a questionnaire and home sleep apnea testing using the WatchPAT-200 device (Itamar-Medical Ltd., Caesarea, Israel), with recording of finger pulse oximetry, actigraphy, body position, peripheral arterial tonometry, and snoring. It has previously been validated using polysomnography [98]. The current analysis included 782 individuals with valid oximetry and Affymetrix 6.0 data.

### Replication group studies

Data from the Sleep Heart Health Study Cardiovascular Health Study (CHS) [99] was available after ARIC and FHS and used in replication analysis. Sleep phenotyping was performed as described earlier. 185 African-American and 731 European-Americans with available polysomnography and Illumina CNV370, and/or Omni1M plus IBC genotypes obtained through dbGaP (pht003699.v1.p1) were analyzed.

The Hispanic Community Health Study/Study of Latinos (HCHS/SOL) is studying risk and protective factors of multiple health conditions in Hispanics/Latinos [100,101]. 16,415 community members from randomly selected households aged 18–74 from 4 cities (Chicago, IL; Miami, FL; Bronx, NY; San Diego, CA) were examined in a baseline exam between 2008–2011. The sample design consisted of a stratified two-stage area probability sample of household addresses. Six cohort backgrounds were represented: Central American (n = 1,730), Cuban (n = 2,348), Dominican (n = 1,460), Mexican (n = 6,471), Puerto-Rican (n = 2,728), and South American (n = 1,068). The exam included anthropometry, questionnaires, and home sleep apnea testing using the ARES Unicorder 5.2 (B-Alert, Carlsbad, CA), which records measurements of airflow using a nasal pressure cannula and pressure transducer; oxyhemoglobin saturation and pulse rate using a forehead-based reflectance oximeter; head movements and position using an accelerometer; and snoring levels using a microphone. The device has undergone previous validation for in-home use [102]. Records were manually scored and cleaned of artifacts at a central sleep reading center [101]. The current study includes 11,351 non-Asian ancestry individuals with oxyhemoglobin saturation values during sleep and Illumina Omni 2.5 genotyping.

The Jackson Heart Study (JHS) is a population-based prospective investigation of cardiovascular disease [103,104]. The ancillary sleep study occurred from 2012–2016, and included home sleep apnea testing with the Embla Embletta Gold, a 6-channel device that includes an oximeter (Broomfield, CO). The device has been validated previously [105]. Additional collected measures include sleep questionnaires and anthropometry. 496 African-American individuals with phenotyping and Affymetrix 6.0 genotyping were included in this study, reflecting a dataset freeze at the time of analysis.

The Western Australian Sleep Health Study (WASHS) is examining the epidemiology and genetics of sleep apnea and related comorbidities [106]. This clinic-based study examines patients presenting to the sole public sleep clinic in Western Australia, located in Perth. 91% of patients were referred for SDB. Data collection for individuals in the current analysis occurred from 2006–2010. In-lab, attended polysomnography was performed using the Compumedics Series E device (Abbotsford, AU). After excluding principal component (PC) outliers (see below), valid oximetry data and genotype data (Illumina Omni 2.5) were available for 1,647 European ancestry patients.

### Phenotype and covariate definitions

The quantitative phenotypic outcome was oxyhemoglobin saturation during sleep (SpO_2_), measured as an average, minimum, or as a percentage of the night with SpO_2_ < 90% (Per90) measured using finger pulse oximetry (all using NONIN oximetry boards) or transcutaneous oximetry (HCHS/SOL only) measured continuously as part of polysomnography or home sleep apnea testing. Other than the WASHS clinical cohort, all sleep data were scored by a central reading center with high levels of established reliability [107] by scorers blinded to all other data. Intermittent waking and SpO_2_ artifact were manually edited from all records. Covariates were obtained by questionnaires, direct measurement (BMI), and oximetry (waking oxygen saturation was measured prior to the sleep recording). Secondary measures such as hemoglobin concentration and spirometry were collected from the same visit whenever possible, however this was not possible for all cohorts (most notably hemoglobin was collected years prior to the sleep exam in some cohorts). Potential device differences were minimized by both performing analyses at a cohort level and using a rank-normal phenotype transformation to reduce the impact of phenotypic outliers. Our analysis focused on identifying potential loci operating in obesity-independent pathways. Hispanic/Latino-specific results have been reported previously for average SpO_2_ [25].

### Genotyping and statistical analyses

Genotypes from all cohorts were imputed to at least a 1000 Genomes Phase 1 density. ARIC, JHS, and HCHS/SOL were imputed using a 1000 Genomes Phase 1 version 3 template. WASHS was imputed using a Haplotype Reference Consortium version 1.0 template [108]. All other cohorts were imputed using a 1000 Genomes Phase 3 version 5 template [109,110]. Single nucleotide polymorphisms (SNPs) and insertions/deletions with minor allele frequency < 0.01, minor allele counts < 20 within a cohort, or an IMPUTE2/PBWT info score < 0.88 were removed from the analysis. Sample sizes and variant counts for each cohort for the three primary phenotype analyses are provided in **S23 Table**.

We explored ancestry-specific associations given past SDB trait differences (*e*.*g*. [93,101]) and linkage disequilibrium differences [109]. Data were analyzed at a cohort- and population-specific level (*e*.*g*. 4 separate analyses for the MESA cohort). Population structure was controlled for using linear mixed models followed by genomic control. Population structure principal components were calculated for the minimally-admixed, self-reported Asian-American and European-American/Australian population groups within individual studies using TRACE [111]. WASHS initial self-reported European ancestry was based on classification of the patient's parents [106]. Individuals were defined as population outliers and removed from analysis if any coordinate from PC 1–4 was greater than 5 standard deviations from the population mean. Individuals self-reporting into groups with modest sample size within a cohort (*e*.*g*. MrOS Asian-Americans) were excluded from study.

Our analysis focused on identifying potential loci operating in obesity-independent pathways. We adjusted for age, age^2^, sex, age × sex, BMI, and BMI^2^ to address known demographic factors and potential non-linear effects of age and BMI. Phenotypes, adjusted for age and sex, were rank-normalized. Residuals were then calculated by further adjusting for BMI. Primary analyses were performed using GEMMA, which incorporates linear mixed models that control for the relatedness structure [112]. HCHS/SOL analyses were performed using the GENESIS Bioconductor package [113] (DOI:10.18129/B9.bioc.GENESIS). A fixed effects, inverse variance weighted meta-analysis was performed using METAL with genomic control applied in each analysis [114]. Variants with p-values < 1 × 10^−6^ in the discovery cohort meta-analysis were carried forward to replication and combined discovery/replication analysis. To reduce the influence of small studies possibly leading to spurious findings, we only present meta-analysis results where 1,000 or more individuals contributed. Individual SNPs in the multi-ethnic analyses were only analyzed if they remained unfiltered in two or more populations. The three traits differed in their final variant counts due to phenotype missingness and ascertainment (no average SpO2 was available for WASHS in this analysis). In aggregate for the discovery cohorts, there were 11,297,250–11,298,080 AA; 8,956,016–8,958,150 EA; and 9,481,040–9,481,751 multi-ethnic variants. The replication cohorts included 12,243,361–12,346,361 AA; 7,448,148–8,707,439 EA; and 10,173,881–10,480,925 multi-ethnic variants. Visualizations were constructed using LocusExplorer and EasyStrata [115,116].

Sleep and sleep disordered breathing may be regulated by multiple tissues [86,117]. Epigenetic database queries were performed using HaploReg version 4 using an imputed model and exact SNPs. HaploReg data included ENCODE and Roadmap Epigenomics consortia data [118–120]. Additional queries examined non-cancerous Blueprint Consortium data (largely related to blood cell lines) and Vermunt *et al*. brain region enhancer data[121–123]. Gene expression data used in expression quantitative trait loci (eQTL) lookups were obtained from multiple studies, including a seven-cohort consortium investigating whole blood (Westra *et al*.) [35,124–130]. The Westra data were pruned to include only eQTL SNPs with FDR < 0.05. Moloc was used to test colocalization [131]. Gene-level analyses used MetaXcan to impute expression levels based on GTEx tissues and Depression and Genes and Networks (DGN) whole blood [132]. GIGSEA, which is designed to work with MetaXcan output, was used for pathway analyses using the whole blood results (queried due to improved power from increased sample size) [133]. We used the weighted linear regression model with 10,000 permutations.

## Supporting information

S1 Fig Average oxygen saturation discovery cohorts manhattan and QQ plots. Top: African-Americans; Middle: European-Americans; Bottom: Multi-ethnic. Variants in the multi-ethnic results had to have results from cohorts in two or more populations.
(PDF)

S2 Fig Minimum oxygen saturation discovery cohorts manhattan and QQ plots. Top: African-Americans; Middle: European-Americans; Bottom: Multi-ethnic (2 or more populations for each variant).
(PDF)

S3 Fig Per90 discovery cohorts manhattan and QQ plots. Top: African-Americans; Middle: European-Americans; Bottom: Multi-ethnic (2 or more populations for each variant).
(PDF)

S4 Fig Multi-ethnic discovery cohorts average oxygen saturation miami plots. Top: Females; Bottom: Males.
(PDF)

S5 Fig Multi-ethnic discovery cohorts minimum oxygen saturation miami plots. Top: Females; Bottom: Males.
(PDF)

S6 Fig Multi-ethnic discovery cohorts Per90 saturation miami plots. Top: Females; Bottom: Males.
(PDF)

S7 Fig Average oxygen saturation regional plot (European ancestry, 10q22). The supplemental regional plot order corresponds to the order found in S2, S3 and S4 Tables, minus the associations shown in Figs 1 and 2.
(PDF)

S8 Fig Per90 regional plot (African-American ancestry, 3p24). (PDF)

S9 Fig Per90 regional plot (African-American ancestry, 4p35). (PDF)

S10 Fig Per90 regional plot (European ancestry, 10q22). (PDF)

S11 Fig Average oxygen saturation regional plot (European ancestry males, 10q22). (PDF)

S12 Fig Average oxygen saturation regional plot (European ancestry males, 12q14). (PDF)

S13 Fig Average oxygen saturation regional plot (European ancestry males, 2p21). (PDF)

S14 Fig Average oxygen saturation regional plot (European ancestry males, 5p15). (PDF)

S15 Fig Average oxygen saturation regional plot (European ancestry males, 5q21). (PDF)

S16 Fig Minimum oxygen saturation regional plot (European ancestry males, 10q22). (PDF)

S17 Fig Minimum oxygen saturation regional plot (European ancestry males, 23p21). (PDF)

S18 Fig Average oxygen saturation regional plot (Combined discovery/replication African-American ancestry, 22q11). (PDF)

S19 Fig Minimum oxygen saturation regional plot (Combined discovery/replication African-American ancestry, 6q25). (PDF)

S20 Fig Minimum oxygen saturation regional plot (Combined discovery/replication European ancestry, 17p13). (PDF)

S21 Fig Minimum oxygen saturation regional plot (Combined discovery/replication European ancestry, 2p12). (PDF)

S22 Fig Per90 regional plot (Combined discovery/replication, 7q22). (PDF)

S1 Table Phenotype and covariate correlations. Spearman correlations between phenotypes and covariates across all discovery cohorts and ethnic groups are shown. Correlations with the spirometry measures FEV1 and FVC are also shown. Correlations were pooled using Fisher Z-transformations weighted by sample size. 95% confidence intervals are listed in parentheses.
(XLS)

S2 Table Significant and suggestive meta-analysis results. Lead SNPs are shown for regions with significant (p < 5.0 × 10–8) and suggestive (p < 1.0 × 10–6) p-values. Discovery regions were carried forward if the combined discovery and replication analysis results indicated a lower p-value, retained genome-level significance, or replication was unavailable (i.e. due to frequency or imputation filters). SNPs denotes the count of regional SNPs with p < 1.0 × 10–6. Genes indicates overlapping Ensembl genes within 5 kb of the p < 1 × 10–6 SNPs. CAF indicates coded allele frequency range. Individual regional SNP results are provided in S3 Table. Individual regions were minimally 500 kb apart.
(XLS)

S3 Table All top locus SNP results (p < 1 x 10–6; full sleep combined-sex and sex-stratified analyses). “Locus Class” indicates “Discovery/Replication” for main analyses or “Combined meta-analysis” for regions not clearing initial discovery p-value thresholds (i.e. S7 Table loci).
(XLS)

S4 Table Sex-stratified comparison of top locus SNPs (p < 1 x 10–6). Sex-stratified samples with n < 1000 are included here for comparison but were not included in the main analyses. An individual variant present in the combined-sex analysis for a given cohort may be missing from the equivalent sex-stratified analysis due to analysis-specific minor allele count thresholds of 20. “Locus Class” indicates “Discovery/Replication” for main analyses or “Combined meta-analysis” for regions not clearing initial discovery p-value thresholds (i.e. S7 Table loci).
(XLS)

S5 Table Lead loci results for other phenotypes. Each lead SNP from the “Original Phenotype” and “Original Model” columns was analyzed using equivalent individuals and models for different phenotypes. Available sample size will vary depending on the availability of the phenotype. AHI values are taken from Chen, Cade, et al. (submitted) “Locus Class” indicates “Discovery/Replication” for main analyses or “Combined meta-analysis” for regions not clearing initial discovery p-value thresholds (i.e. S7 Table loci).
(XLS)

S6 Table Suggestive sex-stratified meta-analysis results. Lead SNPs are shown for regions with suggestive (p < 1.0 × 10–6) p-values. Discovery regions were carried forward if the combined discovery and replication analysis results indicated a lower p-value, retained genome-level significance, or replication was unavailable (i.e. due to frequency or imputation filters). SNPs denotes the count of regional SNPs with p < 1.0 × 10–6. Genes indicates overlapping Ensembl genes within 5 kb of the p < 1 × 10–6 SNPs. CAF indicates coded allele frequency range. Individual regional SNP results are provided in S3 Table. Individual regions were minimally 500 kb apart.
(XLS)

S7 Table Significant combined discovery/replication cohort meta-analysis results. Lead SNPs are shown for regions with significant (p < 5.0 x 10–8) combined-analysis p-values with p > 1 x 10–6 in the discovery phase. SNPs denotes the count of regional SNPs with p < 1.0 x 10–6. Genes indicates overlapping Ensembl genes within 5 kb of the p < 1 x 10–6 SNPs. CAF indicates coded allele frequency range. Individual regional SNP results are provided in S3 Table. Individual regions were minimally 500 kb apart.
(XLS)

S8 Table Lead loci results adjusted for additional covariates. Lead variants were re-analyzed with standard models and additional covariates as listed. As these covariates were not collected in all cohorts and individuals, the analysis were also performed with the same individuals for direct comparisons (matched N control columns).
(XLS)

S9 Table Top locus SNP (p < 1 x 10–6) HaploReg (Roadmap Epigenomics and ENCODE) epigenetic evidence. Data were obtained from HaploReg 4.1 (https://pubs.broadinstitute.org/mammals/haploreg/haploreg.php) using exact SNPs (LD threshold = NA) and a ChromHMM 15-state model, followed by extraction from HTML. “Locus Class” indicates “Discovery/Replication” for main analyses or “Combined meta-analysis” for regions not clearing initial discovery p-value thresholds (i.e. S7 Table loci).
(XLS)

S10 Table Top locus SNP (p < 1 x 10–6) Blueprint and Vermunt epigenetic evidence. Blueprint cell lines are a non-cancerous sub-set of largely blood cells. Vermunt brain region samples were not assayed for promoter regions. “Locus Class” indicates “Discovery/Replication” for main analyses or “Combined meta-analysis” for regions not clearing initial discovery p-value thresholds (i.e. S7 Table loci).
(XLS)

S11 Table Top locus SNP (p < 1 x 10–6) eQTL evidence. Data were obtained from Fairfax et al. (monocytes), Geuvadis (LCLs), GTEx, Hao et al. (lung), Muther (adipose, LCLs, skin), Raj et al. (CD4, monocytes), Westra et al. (whole blood), and Zeller et al. (monocytes). “Locus Class” indicates “Discovery/Replication” for main analyses or “Combined meta-analysis” for regions not clearing initial discovery p-value thresholds (i.e. S7 Table loci).
(XLS)

S12 Table Multi-variate MTAG (p < 10 x 10–6) results. MTAG analysis was performed on European ancestry samples using our summary statistics for the three traits simultaneously. Lead results (p < 1 x 10–6) are shown.
(XLS)

S13 Table MetaXcan gene-level results for subcutaneous adipose tissue in European-Americans. Lead results (p < 0.05) are shown. Note that Ensembl IDs obtained from MetaXcan are occasionally unavailable.
(XLS)

S14 Table MetaXcan gene-level results for visceral omentum adipose tissue in European-Americans. Lead results (p < 0.05) are shown. Note that Ensembl IDs obtained from MetaXcan are occasionally unavailable.
(XLS)

S15 Table MetaXcan gene-level results for hypothalamus in European-Americans. Lead results (p < 0.05) are shown. Note that Ensembl IDs obtained from MetaXcan are occasionally unavailable.
(XLS)

S16 Table MetaXcan gene-level results for liver in European-Americans. Lead results (p < 0.05) are shown. Note that Ensembl IDs obtained from MetaXcan are occasionally unavailable.
(XLS)

S17 Table MetaXcan gene-level results for lung in European-Americans. Lead results (p < 0.05) are shown. Note that Ensembl IDs obtained from MetaXcan are occasionally unavailable.
(XLS)

S18 Table MetaXcan gene-level results for skeletal muscle in European-Americans. Lead results (p < 0.05) are shown. Note that Ensembl IDs obtained from MetaXcan are occasionally unavailable.
(XLS)

S19 Table MetaXcan gene-level results for DGN whole blood in European-Americans. Lead results (p < 0.05) are shown. Note that Ensembl IDs obtained from MetaXcan are occasionally unavailable.
(XLS)

S20 Table GIGSEA KEGG pathway enrichment in whole blood analyses. MetaXcan gene-level results for DGN whole blood (S19 Table, all p-values) were used as input.
(XLS)

S21 Table GIGSEA micro RNA enrichment in whole blood analyses. MetaXcan gene-level results for DGN whole blood (S19 Table, all p-values) were used as input. MicroRNAs are curated by the Molecular Signatures Database (http://software.broadinstitute.org/gsea/msigdb/index.jsp).
(XLS)

S22 Table GIGSEA transcription factor binding site enrichment in whole blood analyses. MetaXcan gene-level results for DGN whole blood (S19 Table, all p-values) were used as input. Transcription factor binding sites are curated by the Molecular Signatures Database (http://software.broadinstitute.org/gsea/msigdb/index.jsp).
(XLS)

S23 Table Study-level variant counts. Cohort-level information is shown for the three primary analyses, including discovery/replication category, the number of individuals with available phenotyping and genotyping, genotyping platform, imputation panel, and the number of imputed variants tested in each analysis following info score, MAC, and MAF filtering.
(XLS)
