# Supplementary material for: Associations of variants In the hexokinase 1 and interleukin 18 receptor regions with oxyhemoglobin saturation during sleep
Source: PLoS Genet. 2019 Apr 16;15(4):e1007739. doi: 10.1371/journal.pgen.1007739 (PMC6467367; doi:10.1371/journal.pgen.1007739)
Supplement: S1 Fig — Top: African-Americans; Middle: European-Americans; Bottom: Multi-ethnic. Variants in the multi-ethnic results had to have results from cohorts in two or more populations. (PDF) [file pgen.1007739.s001.pdf]

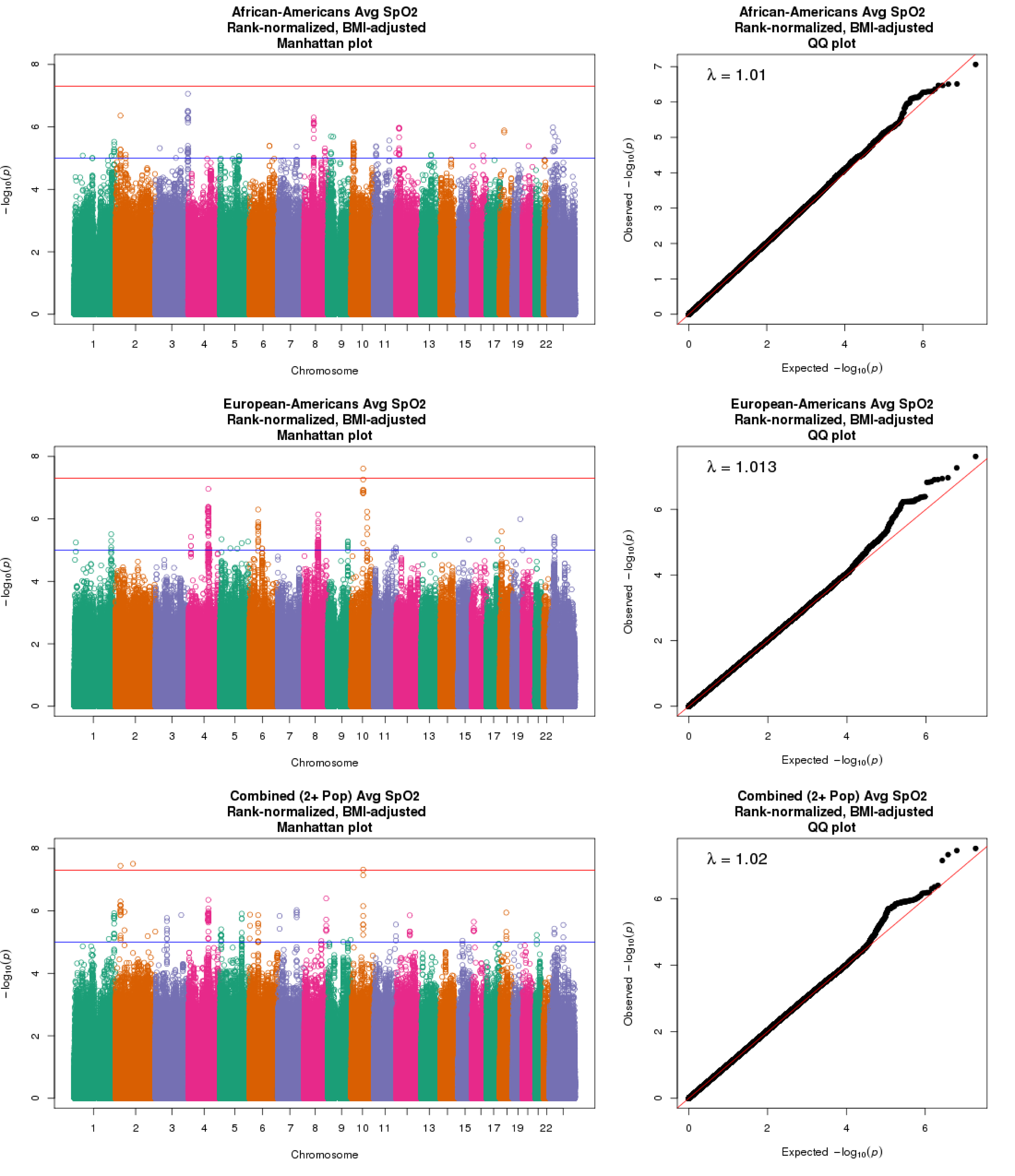
African-Americans Avg SpO2
Rank-normalized, BMI-adjusted
Manhattan plot
Chromosome
−log10(p)
African-Americans Avg SpO2
Rank-normalized, BMI-adjusted
QQ plot
λ = 1.01
Expected −log10(p)
Observed −log10(p)
European-Americans Avg SpO2
Rank-normalized, BMI-adjusted
Manhattan plot
Chromosome
−log10(p)
European-Americans Avg SpO2
Rank-normalized, BMI-adjusted
QQ plot
λ = 1.013
Expected −log10(p)
Observed −log10(p)
Combined (2+ Pop) Avg SpO2
Rank-normalized, BMI-adjusted
Manhattan plot
Chromosome
−log10(p)
Combined (2+ Pop) Avg SpO2
Rank-normalized, BMI-adjusted
QQ plot
λ = 1.02
Expected −log10(p)
Observed −log10(p)
