# Supplementary material for: Associations of variants In the hexokinase 1 and interleukin 18 receptor regions with oxyhemoglobin saturation during sleep
Source: PLoS Genet. 2019 Apr 16;15(4):e1007739. doi: 10.1371/journal.pgen.1007739 (PMC6467367; doi:10.1371/journal.pgen.1007739)
Supplement: S2 Fig — Top: African-Americans; Middle: European-Americans; Bottom: Multi-ethnic (2 or more populations for each variant). (PDF) [file pgen.1007739.s002.pdf]

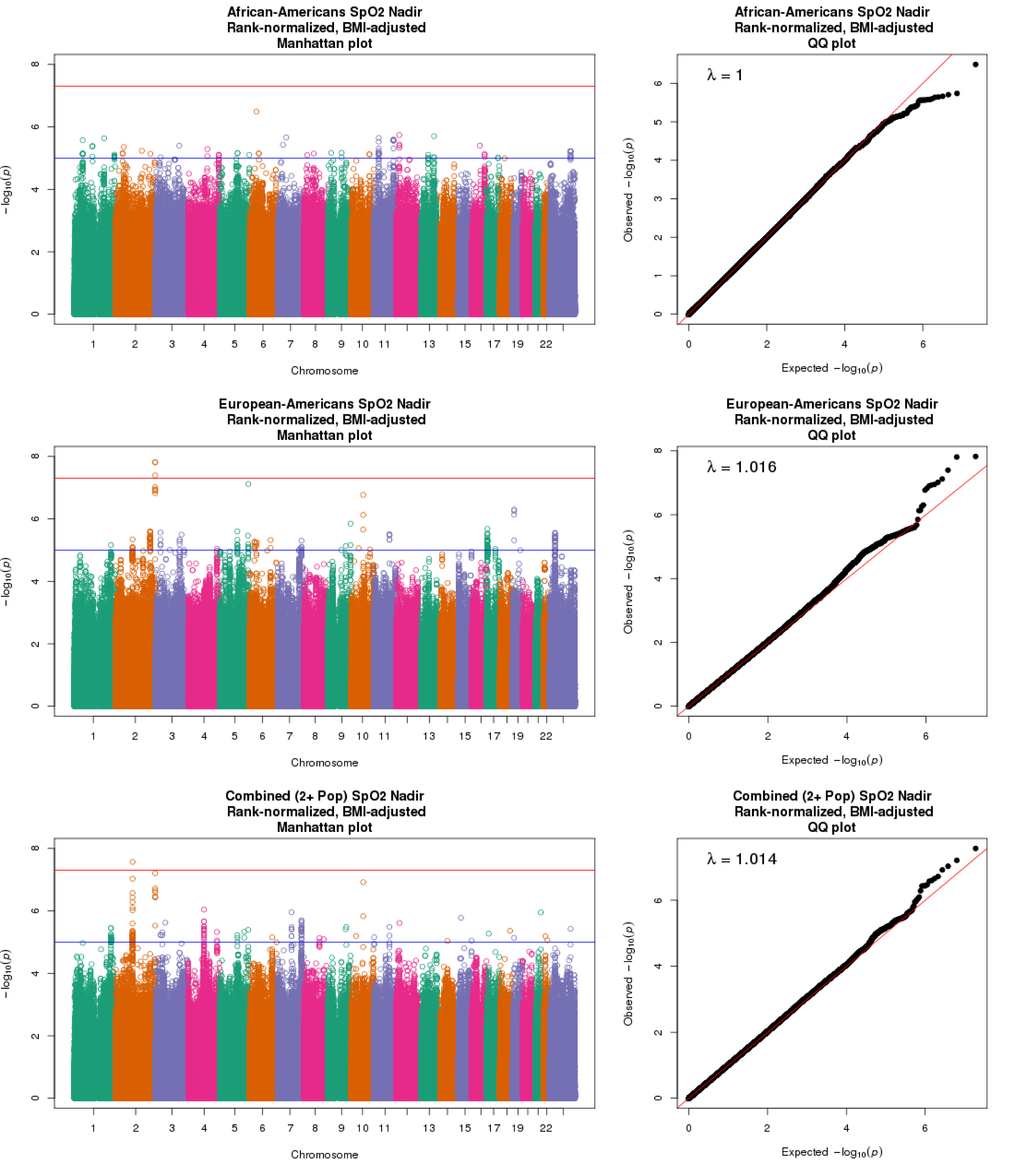
African-Americans SpO2 Nadir
Rank-normalized, BMI-adjusted
Manhattan plot
−log10(p)
Chromosome
1 2 3 4 5 6 7 8 9 10 11 13 15 17 19 22
African-Americans SpO2 Nadir
Rank-normalized, BMI-adjusted
QQ plot
λ = 1
Observed −log10(p)
Expected −log10(p)
European-Americans SpO2 Nadir
Rank-normalized, BMI-adjusted
Manhattan plot
−log10(p)
Chromosome
1 2 3 4 5 6 7 8 9 10 11 13 15 17 19 22
European-Americans SpO2 Nadir
Rank-normalized, BMI-adjusted
QQ plot
λ = 1.016
Observed −log10(p)
Expected −log10(p)
Combined (2+ Pop) SpO2 Nadir
Rank-normalized, BMI-adjusted
Manhattan plot
−log10(p)
Chromosome
1 2 3 4 5 6 7 8 9 10 11 13 15 17 19 22
Combined (2+ Pop) SpO2 Nadir
Rank-normalized, BMI-adjusted
QQ plot
λ = 1.014
Observed −log10(p)
Expected −log10(p)
