# Supplementary material for: Associations of variants In the hexokinase 1 and interleukin 18 receptor regions with oxyhemoglobin saturation during sleep
Source: PLoS Genet. 2019 Apr 16;15(4):e1007739. doi: 10.1371/journal.pgen.1007739 (PMC6467367; doi:10.1371/journal.pgen.1007739)
Supplement: S3 Fig — Top: African-Americans; Middle: European-Americans; Bottom: Multi-ethnic (2 or more populations for each variant). (PDF) [file pgen.1007739.s003.pdf]

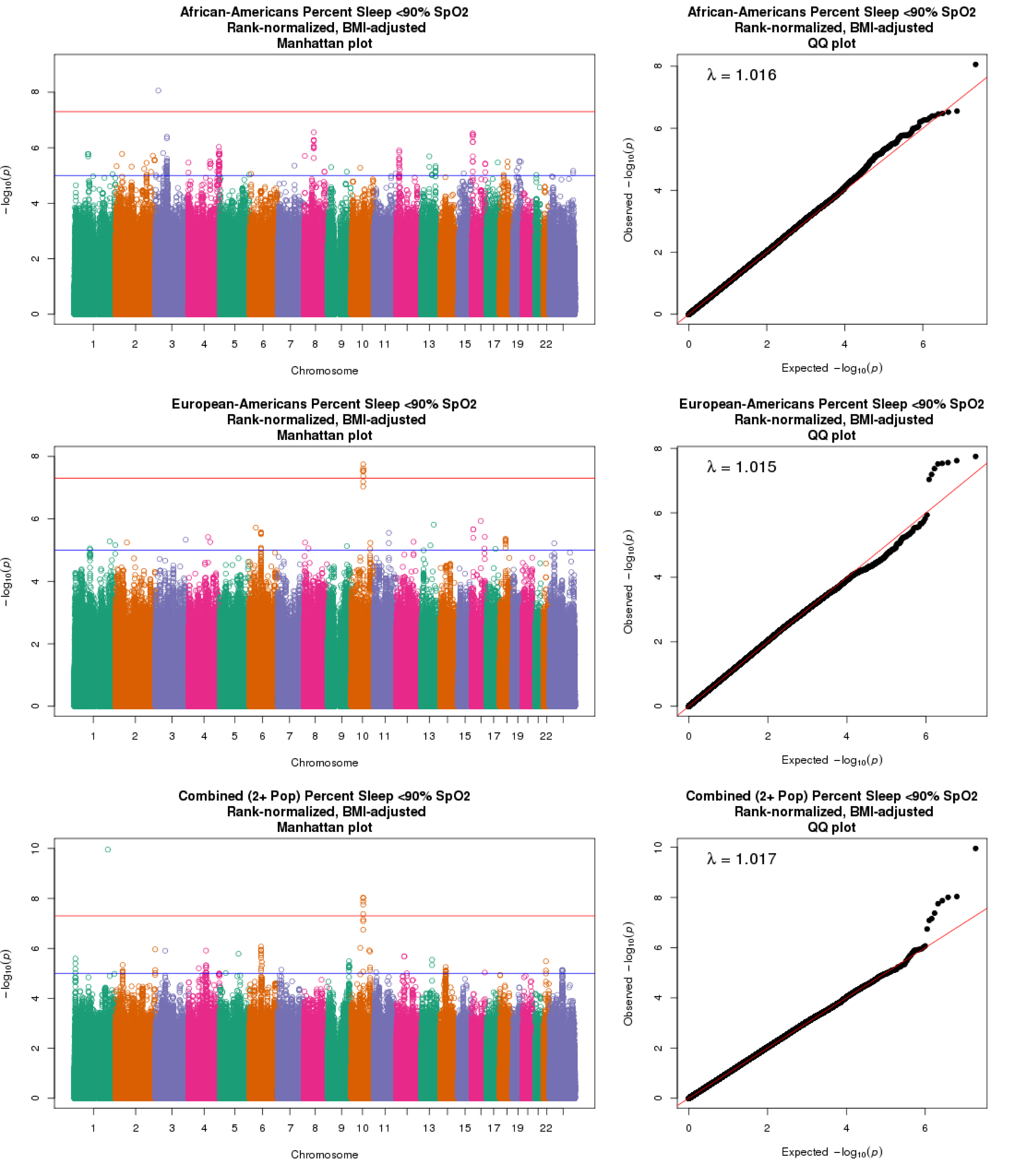
African-Americans Percent Sleep <90% SpO2
Rank-normalized, BMI-adjusted
Manhattan plot
Chromosome
African-Americans Percent Sleep <90% SpO2
Rank-normalized, BMI-adjusted
QQ plot
λ = 1.016
Expected −log10(p)
Observed −log10(p)
European-Americans Percent Sleep <90% SpO2
Rank-normalized, BMI-adjusted
Manhattan plot
Chromosome
European-Americans Percent Sleep <90% SpO2
Rank-normalized, BMI-adjusted
QQ plot
λ = 1.015
Expected −log10(p)
Observed −log10(p)
Combined (2+ Pop) Percent Sleep <90% SpO2
Rank-normalized, BMI-adjusted
Manhattan plot
Chromosome
Combined (2+ Pop) Percent Sleep <90% SpO2
Rank-normalized, BMI-adjusted
QQ plot
λ = 1.017
Expected −log10(p)
Observed −log10(p)
