# Supplementary figures and images for: Associations of variants In the hexokinase 1 and interleukin 18 receptor regions with oxyhemoglobin saturation during sleep
Source: PLoS Genet. 2019 Apr 16;15(4):e1007739. doi: 10.1371/journal.pgen.1007739 (PMC6467367; doi:10.1371/journal.pgen.1007739)

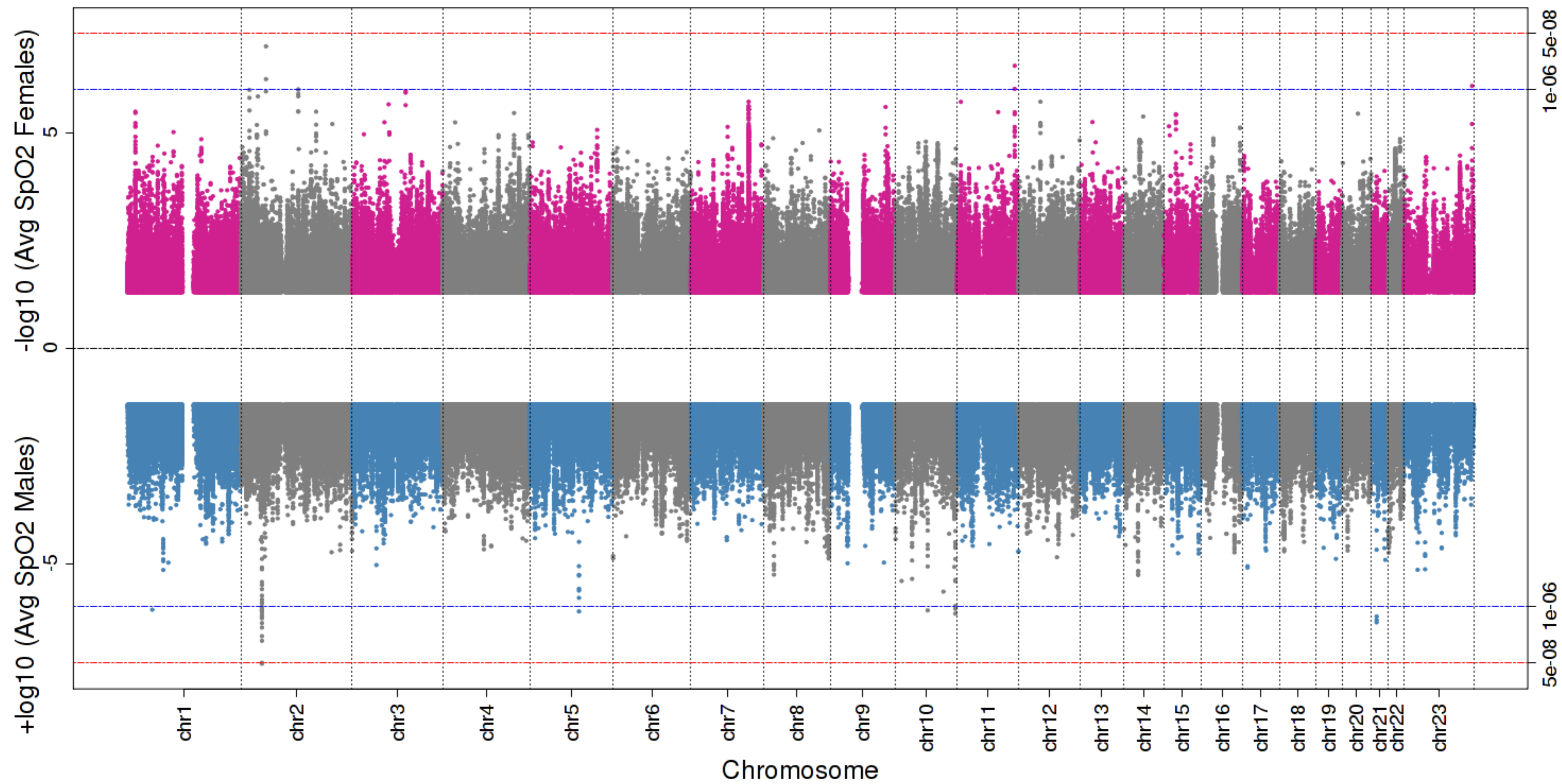

Supplement: S4 Fig — Top: Females; Bottom: Males. (PDF) [file pgen.1007739.s004.pdf]

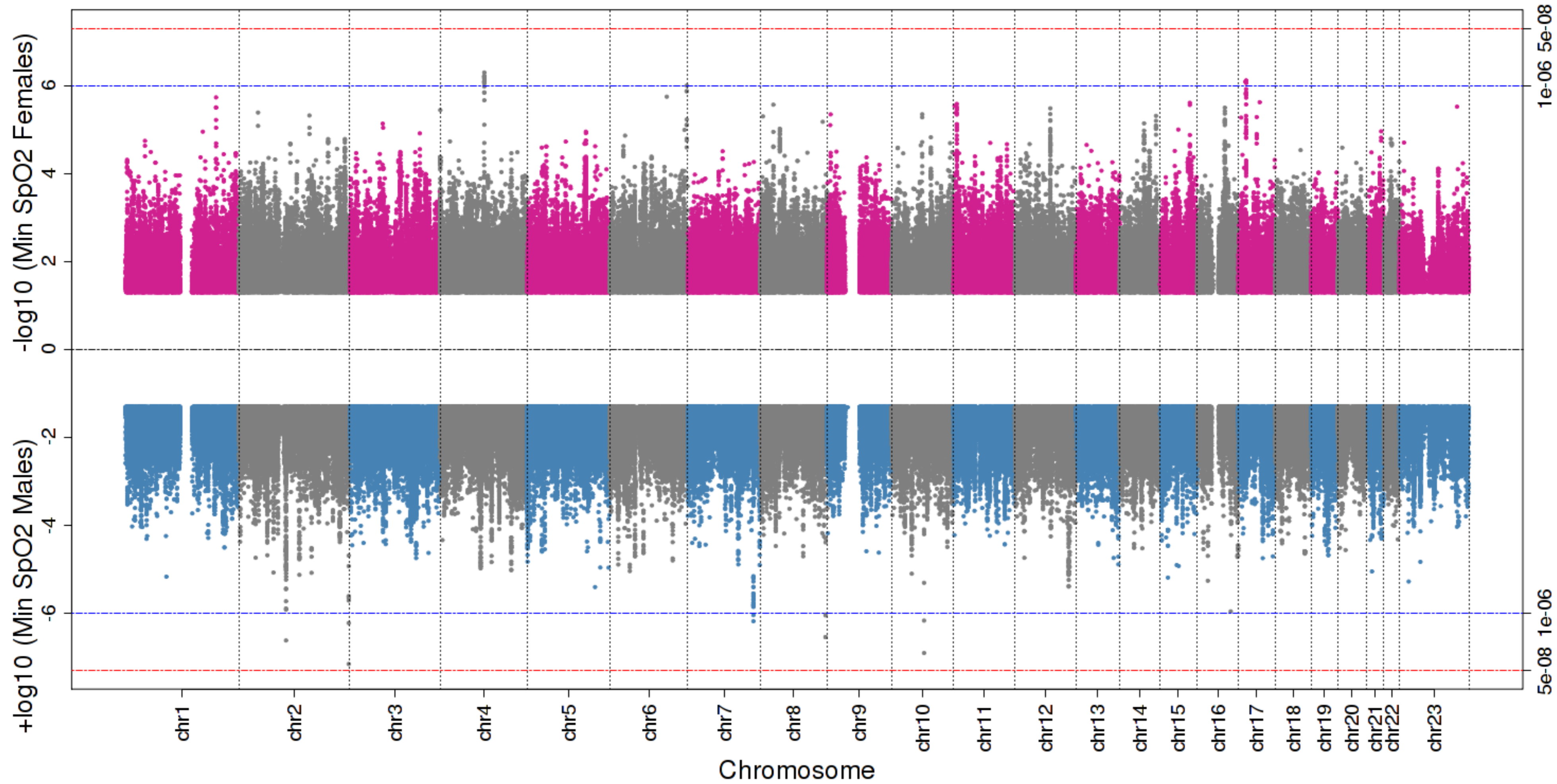

Supplement: S5 Fig — Top: Females; Bottom: Males. (PDF) [file pgen.1007739.s005.pdf]

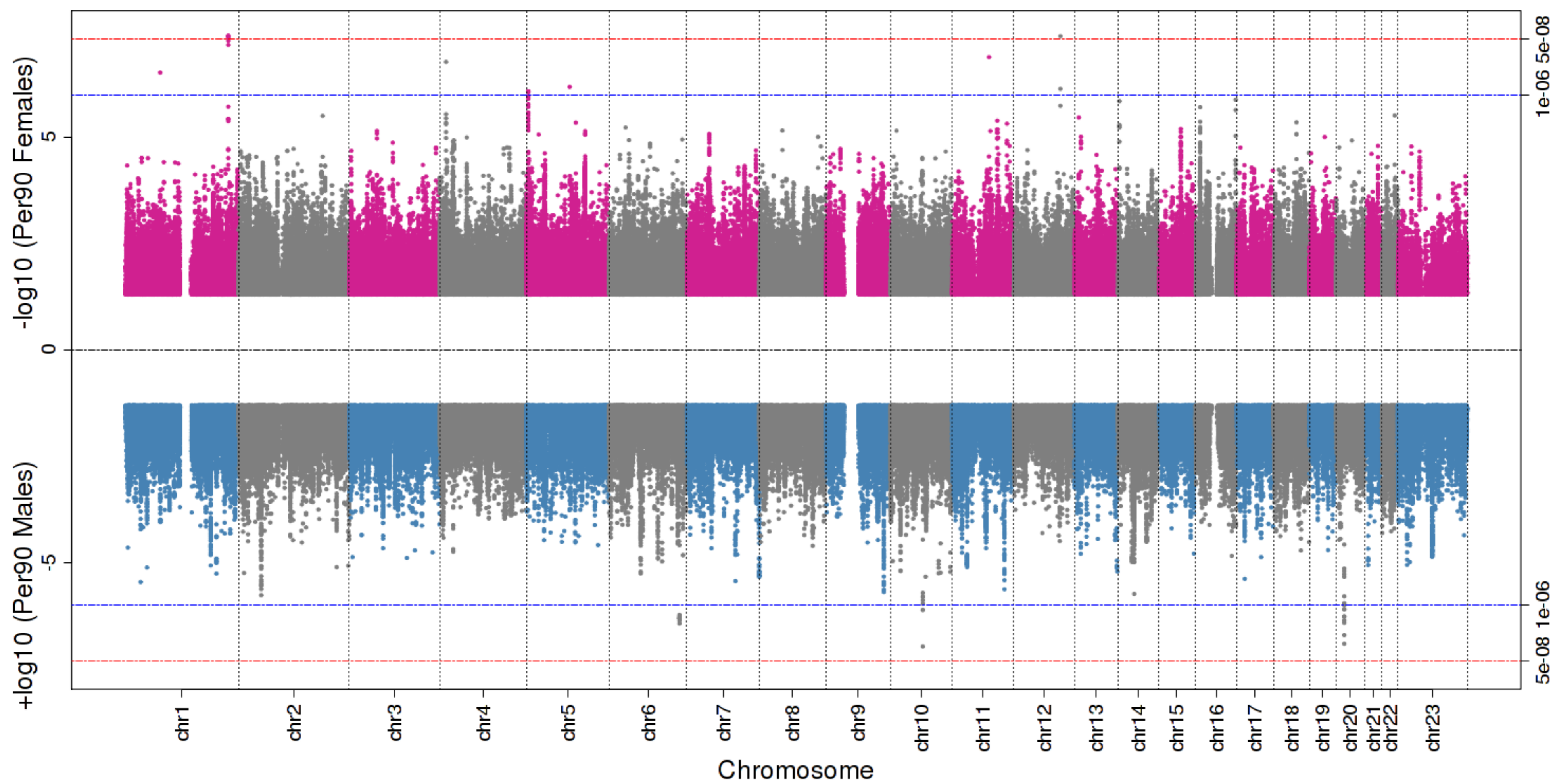

Supplement: S6 Fig — Top: Females; Bottom: Males. (PDF) [file pgen.1007739.s006.pdf]

Figure S7 Avg SpO2 EA 10q22 rs16926246

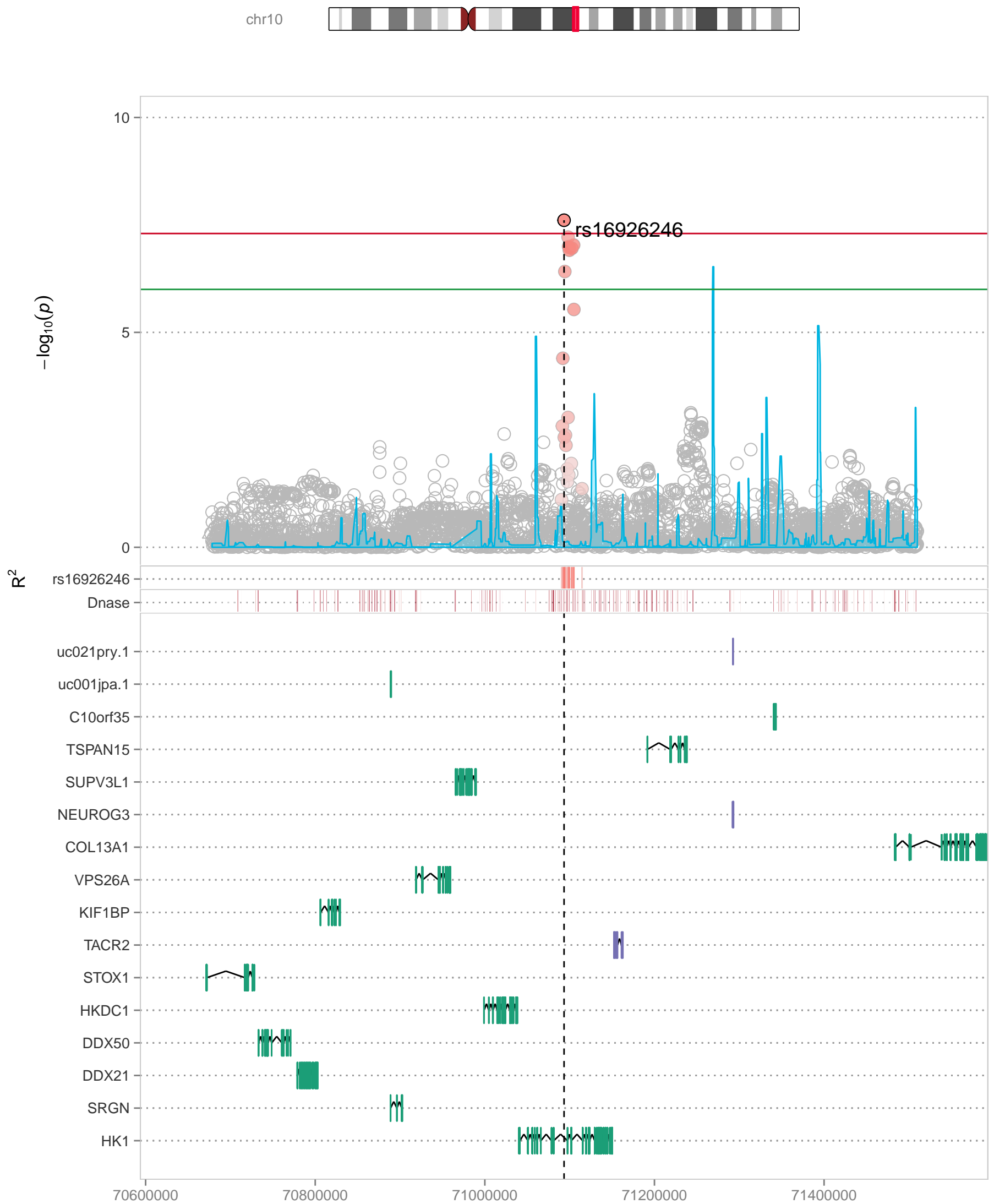

Supplement: S7 Fig — The supplemental regional plot order corresponds to the order found in S2, S3 and S4 Tables, minus the associations shown in Figs 1 and 2. (PDF) [file pgen.1007739.s007.pdf]

Figure S8 Per90 AA 3p24 rs17042395

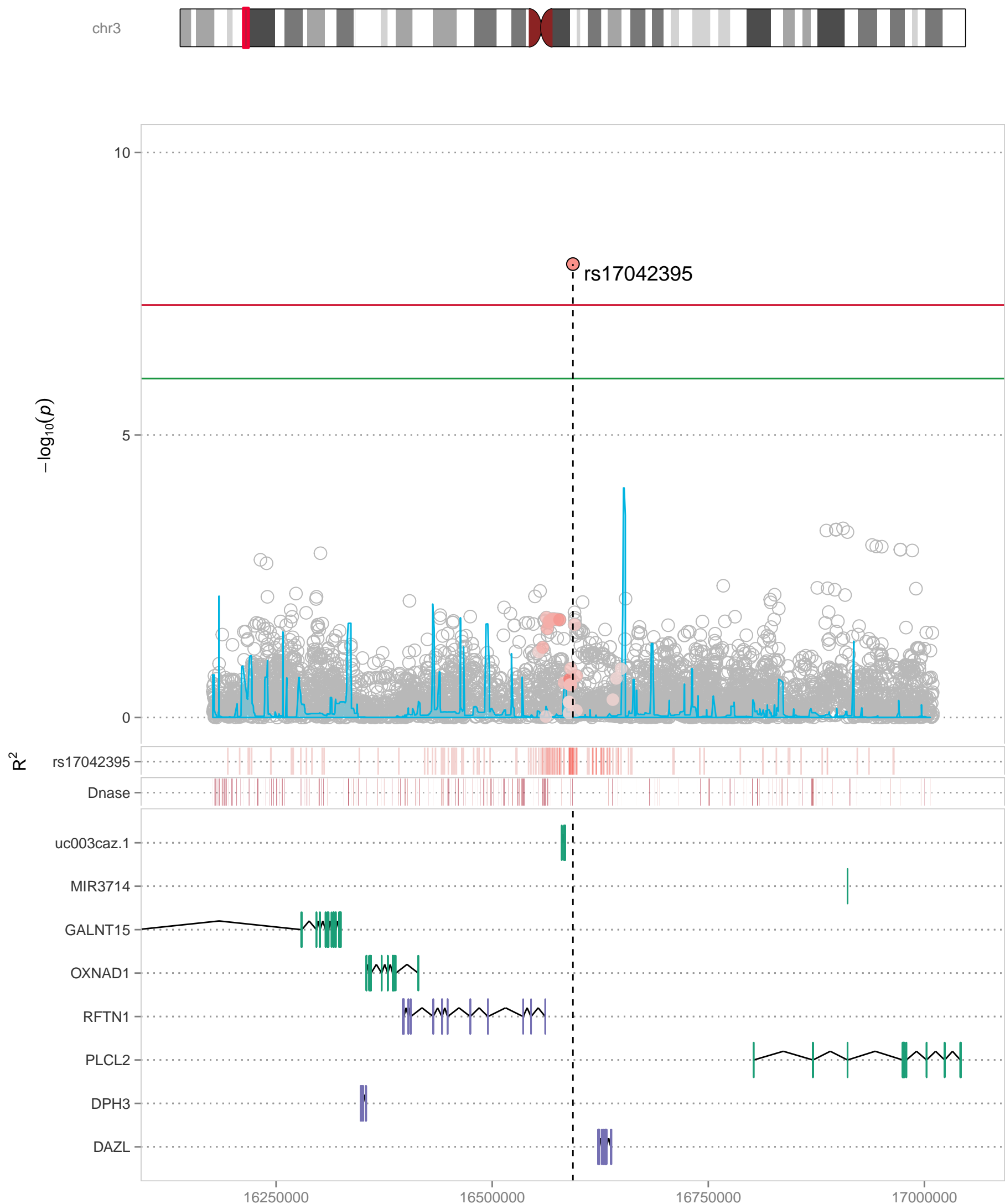

Supplement: S8 Fig — (PDF) [file pgen.1007739.s008.pdf]

Figure S9 Per90 AA 4q35 rs561718421

chr4

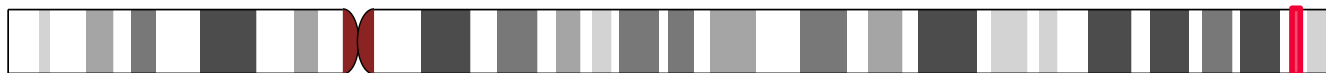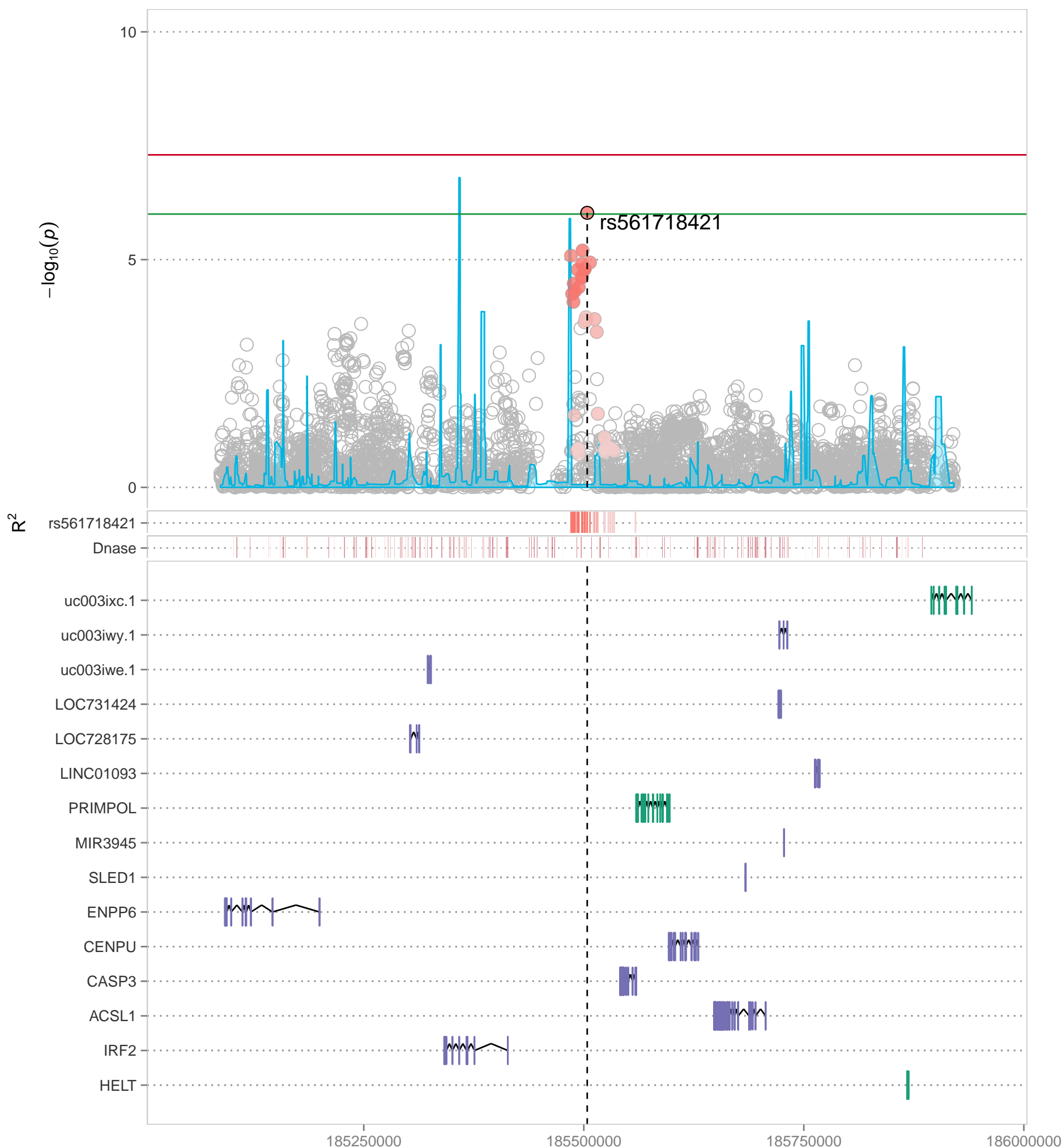

Supplement: S9 Fig — (PDF) [file pgen.1007739.s009.pdf]

Figure S10 Per90 EA 10q22 rs148471505

chr10

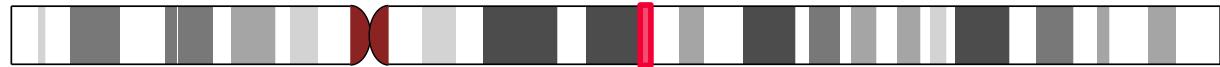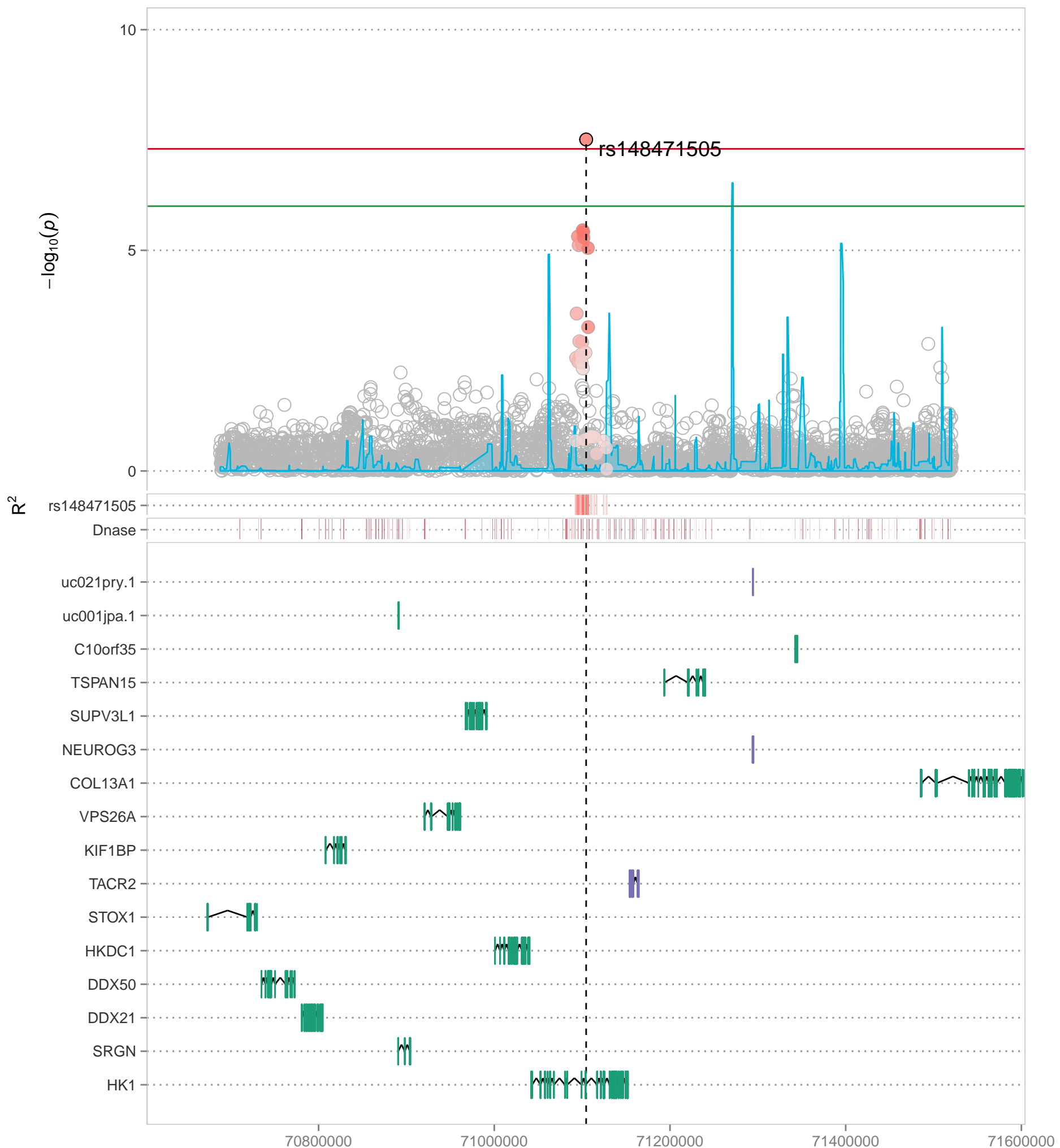

Supplement: S10 Fig — (PDF) [file pgen.1007739.s010.pdf]

Figure S11 Avg SpO2 EA Males 18p11 rs78805840

chr18

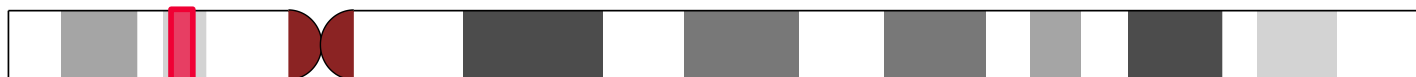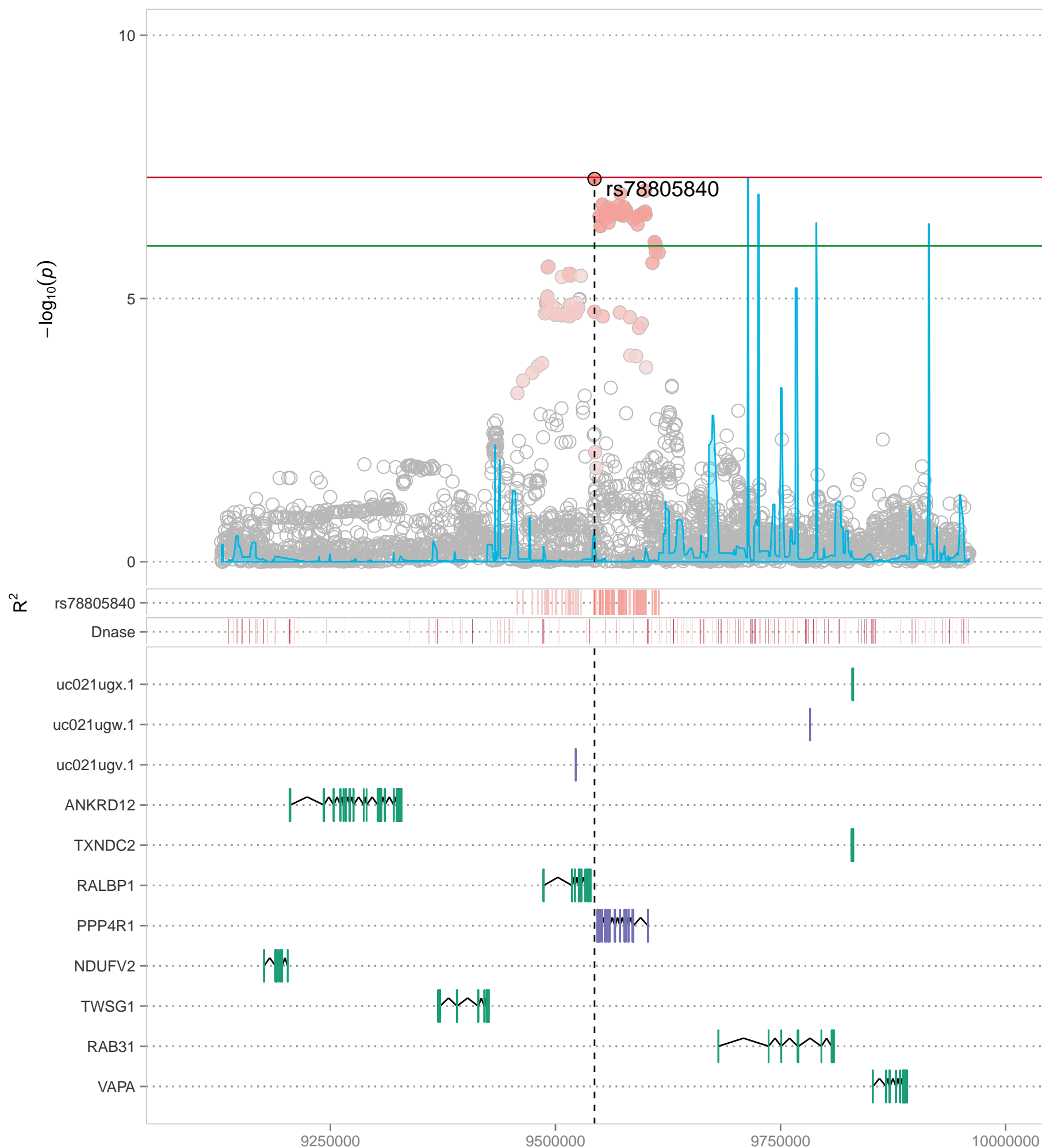

Supplement: S11 Fig — (PDF) [file pgen.1007739.s011.pdf]

Figure S12 Avg SpO2 EA Males 12q14 rs117294696

chr12

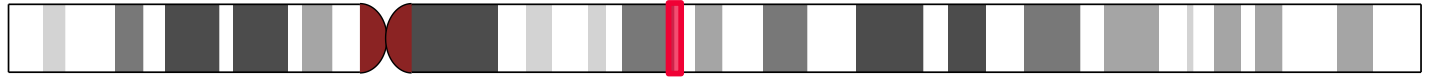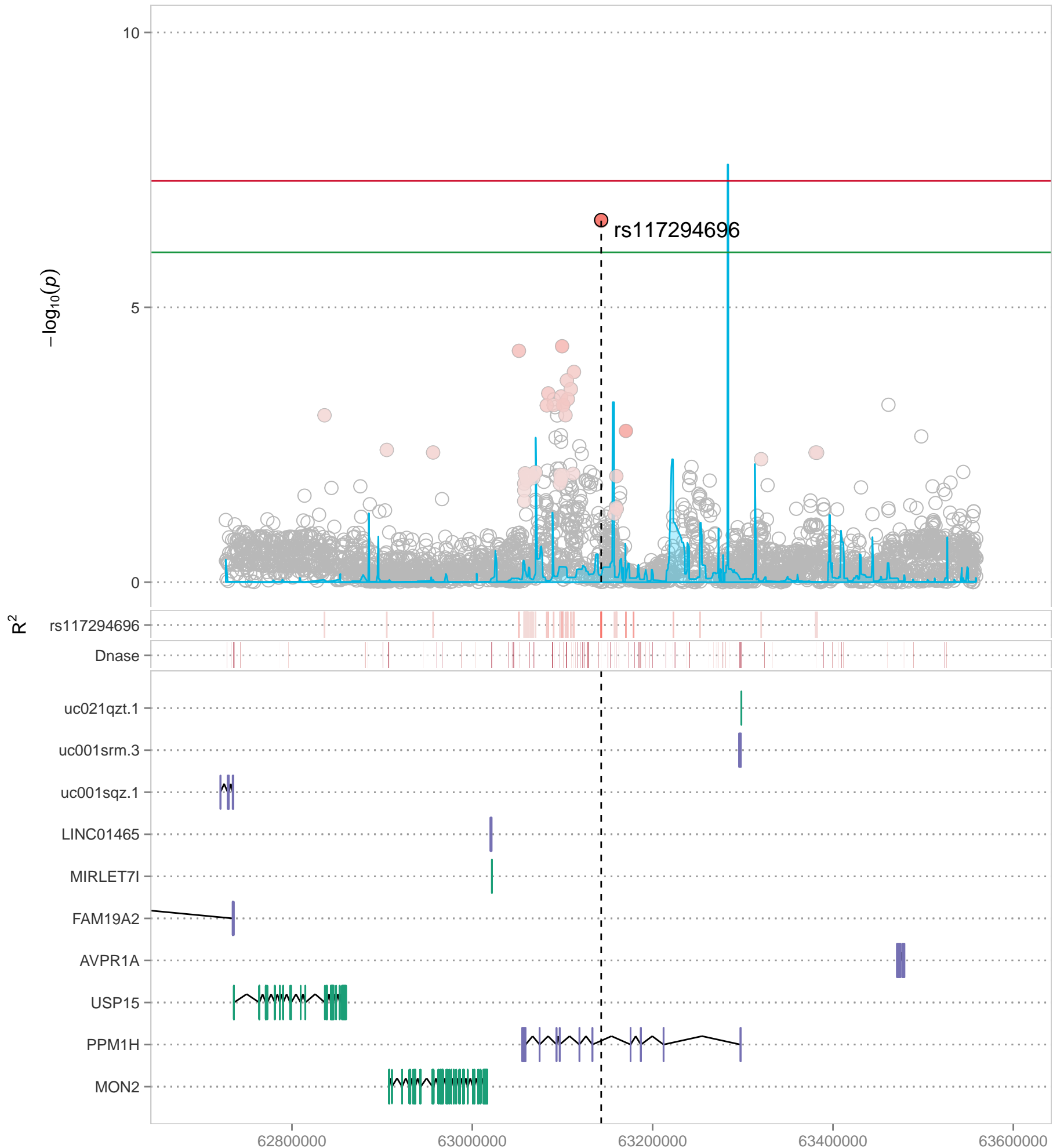

Supplement: S12 Fig — (PDF) [file pgen.1007739.s012.pdf]

Figure S13 Avg SpO2 EA Males 2p21 rs7578254

chr2

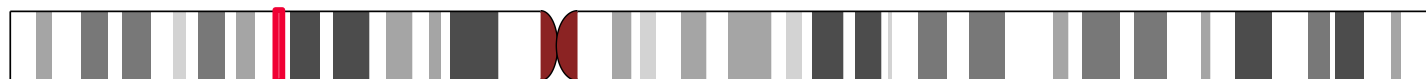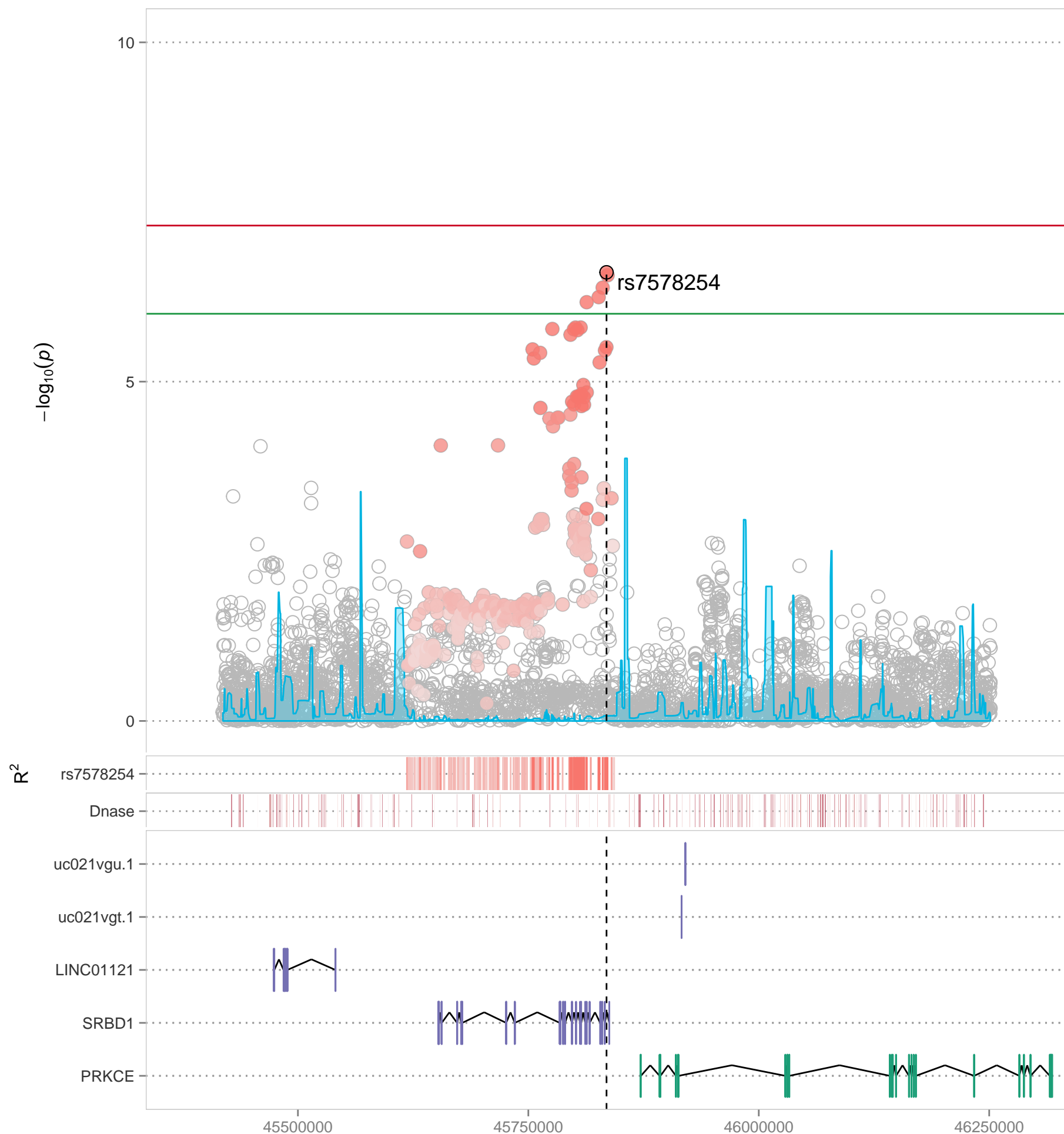

Supplement: S13 Fig — (PDF) [file pgen.1007739.s013.pdf]

Figure S14 Avg SpO2 EA Males 5p15 rs852576

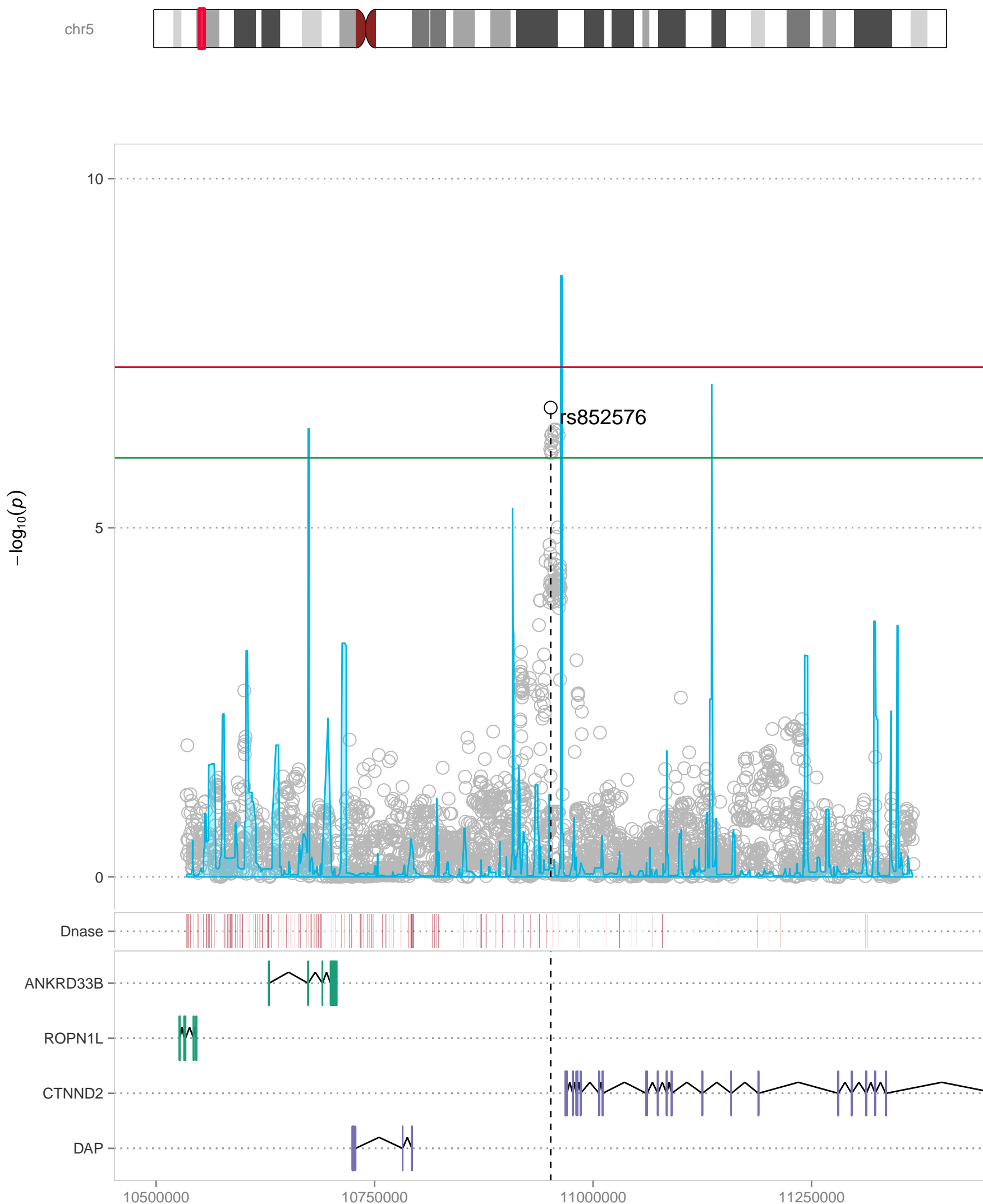

Supplement: S14 Fig — (PDF) [file pgen.1007739.s014.pdf]

Figure S15 Avg SpO2 EA Males 5q21 rs17594657

chr5

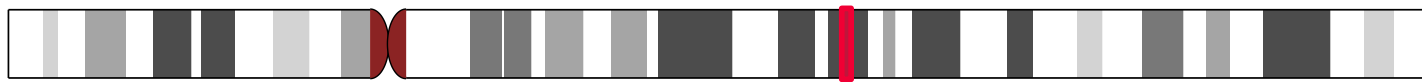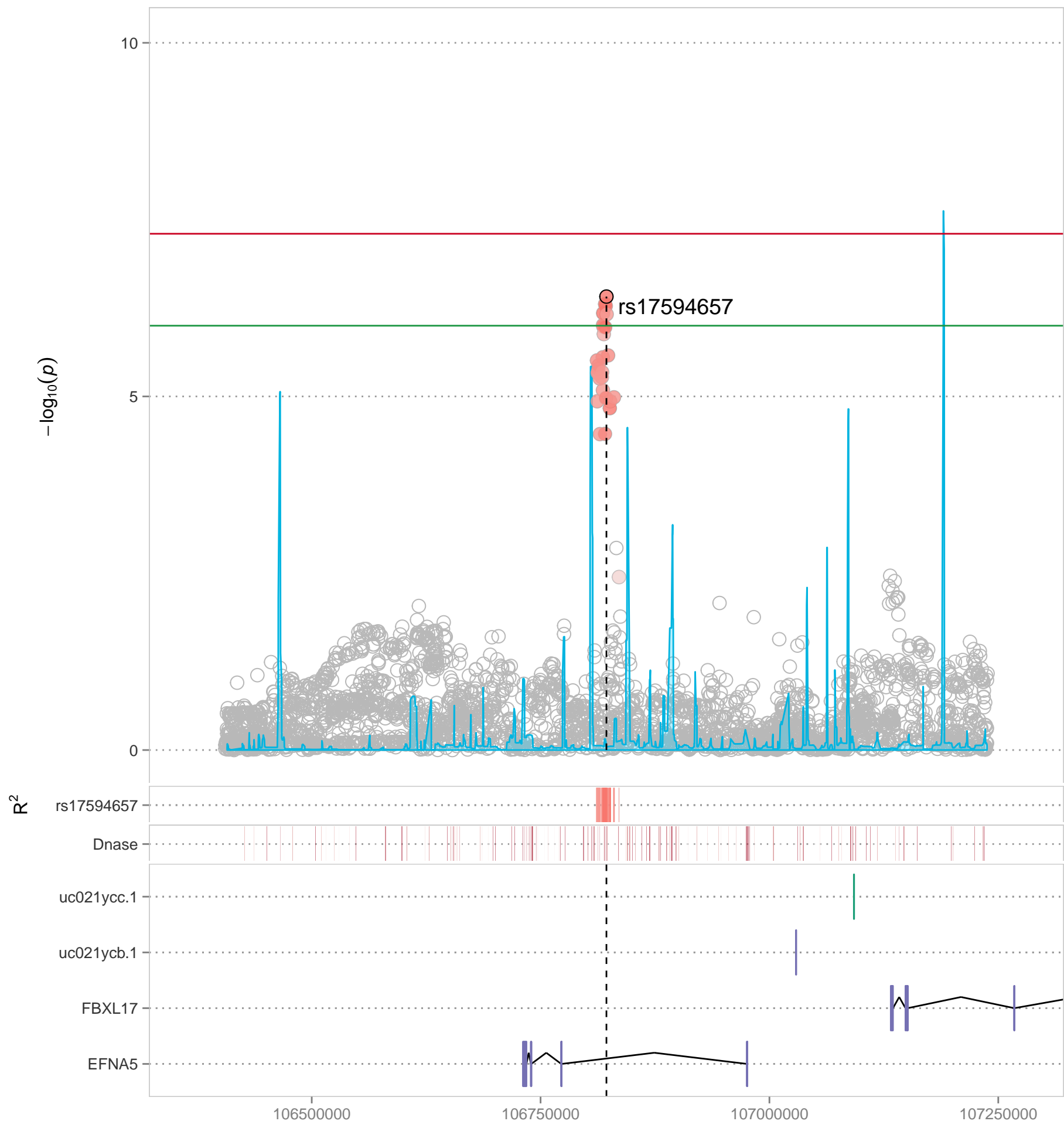

Supplement: S15 Fig — (PDF) [file pgen.1007739.s015.pdf]

Figure S16 Min SpO2 EA Males 10q22 rs17476364

chr10

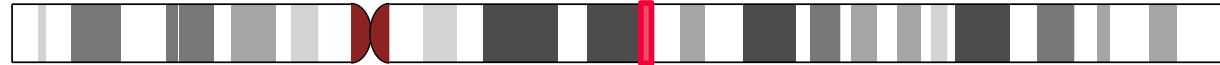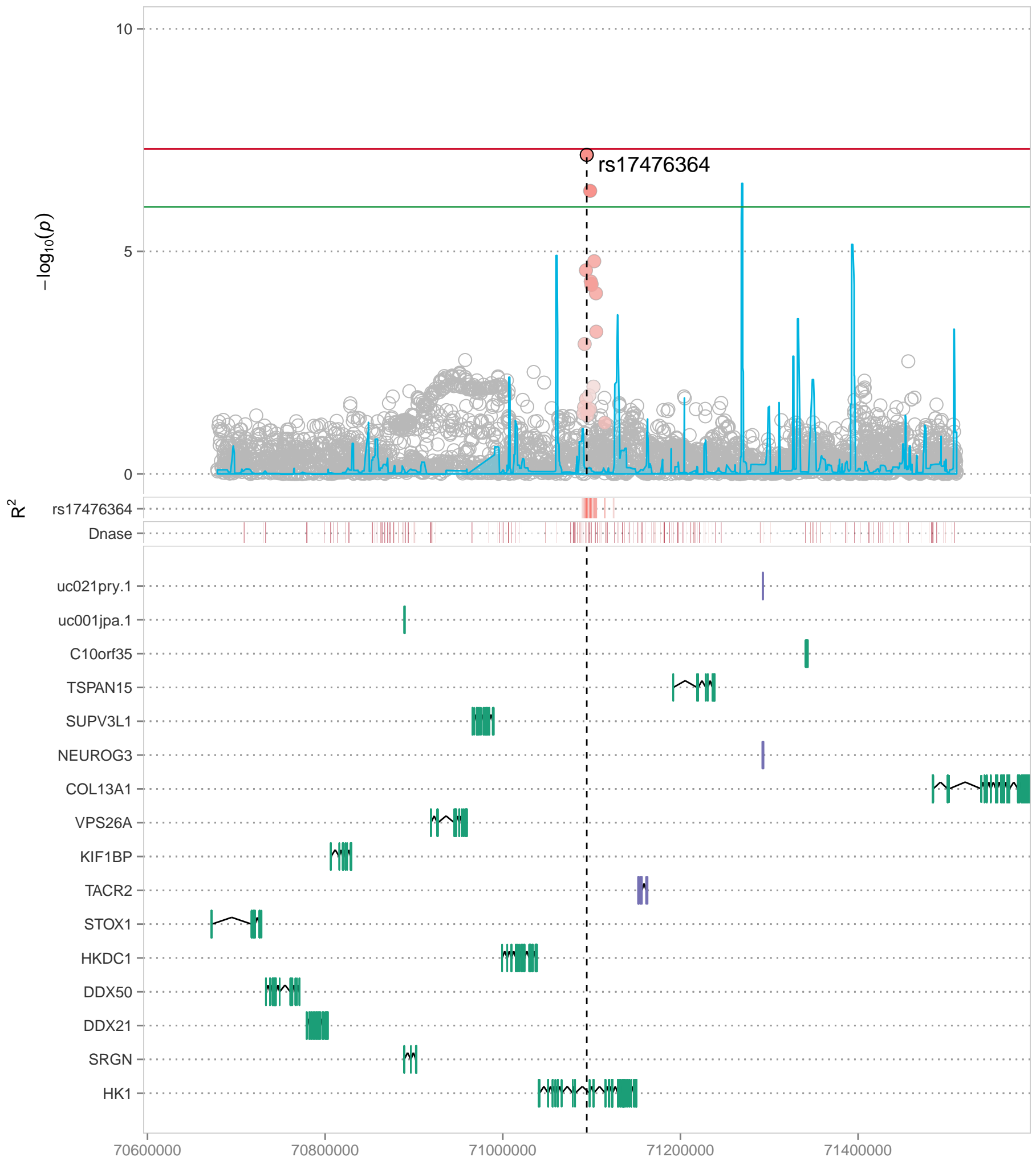

Supplement: S16 Fig — (PDF) [file pgen.1007739.s016.pdf]

Fig S17 Per90 EA Males 23p21 rs5928424

Plotted SNPs

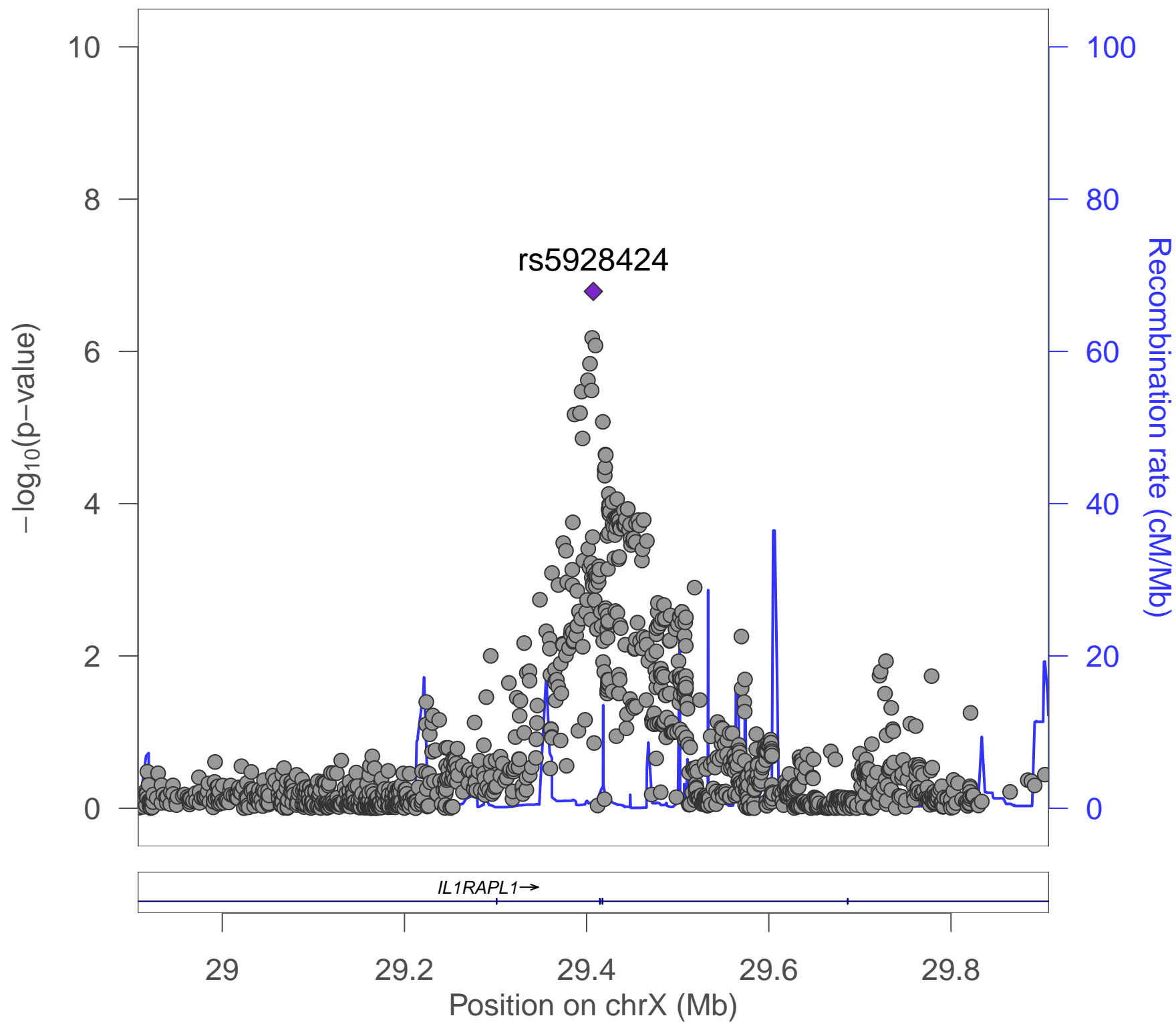

Supplement: S17 Fig — (PDF) [file pgen.1007739.s017.pdf]

Figure S18 Avg SpO2 AA 22q11 rs74822035

chr22

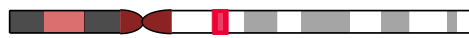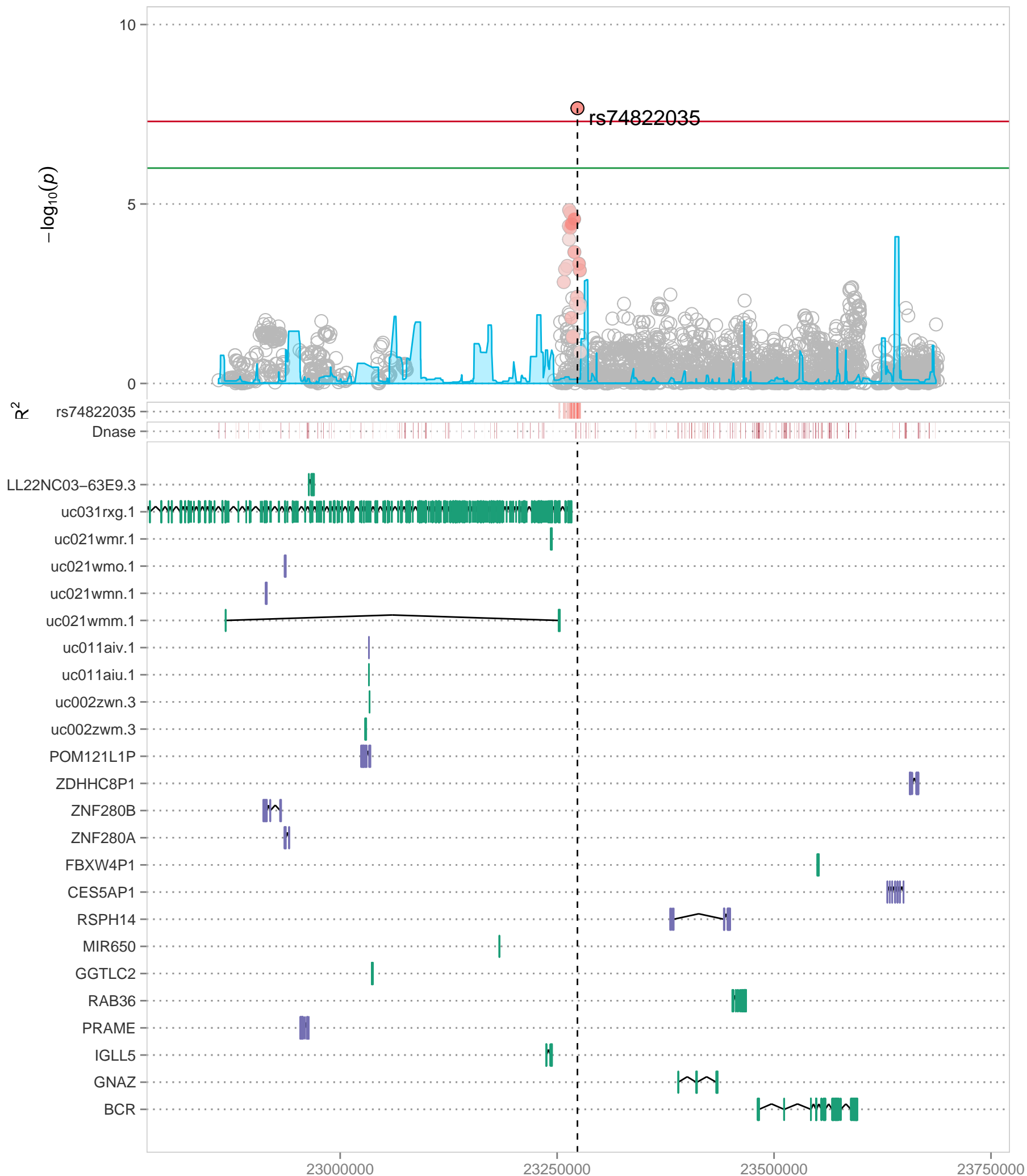

Supplement: S18 Fig — (PDF) [file pgen.1007739.s018.pdf]

Figure S19 Min SpO2 AA 6q25 rs116614712

chr6

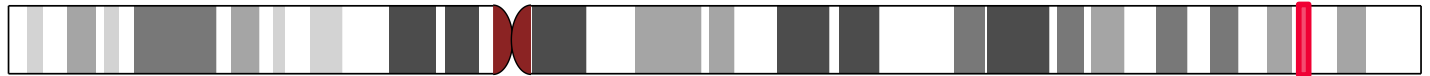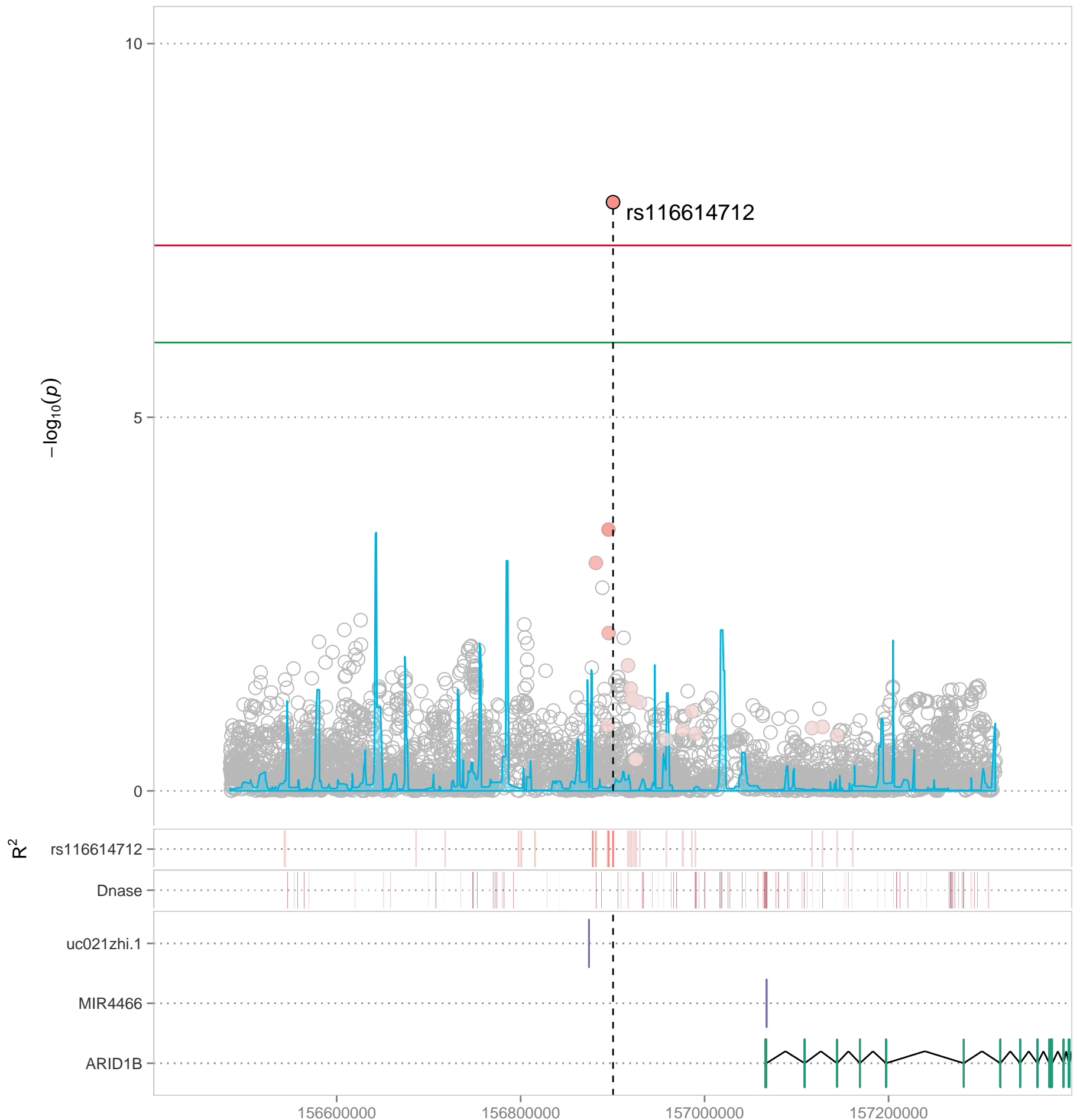

Supplement: S19 Fig — (PDF) [file pgen.1007739.s019.pdf]

Figure S20 Min SpO2 EA 17p13 rs12150370

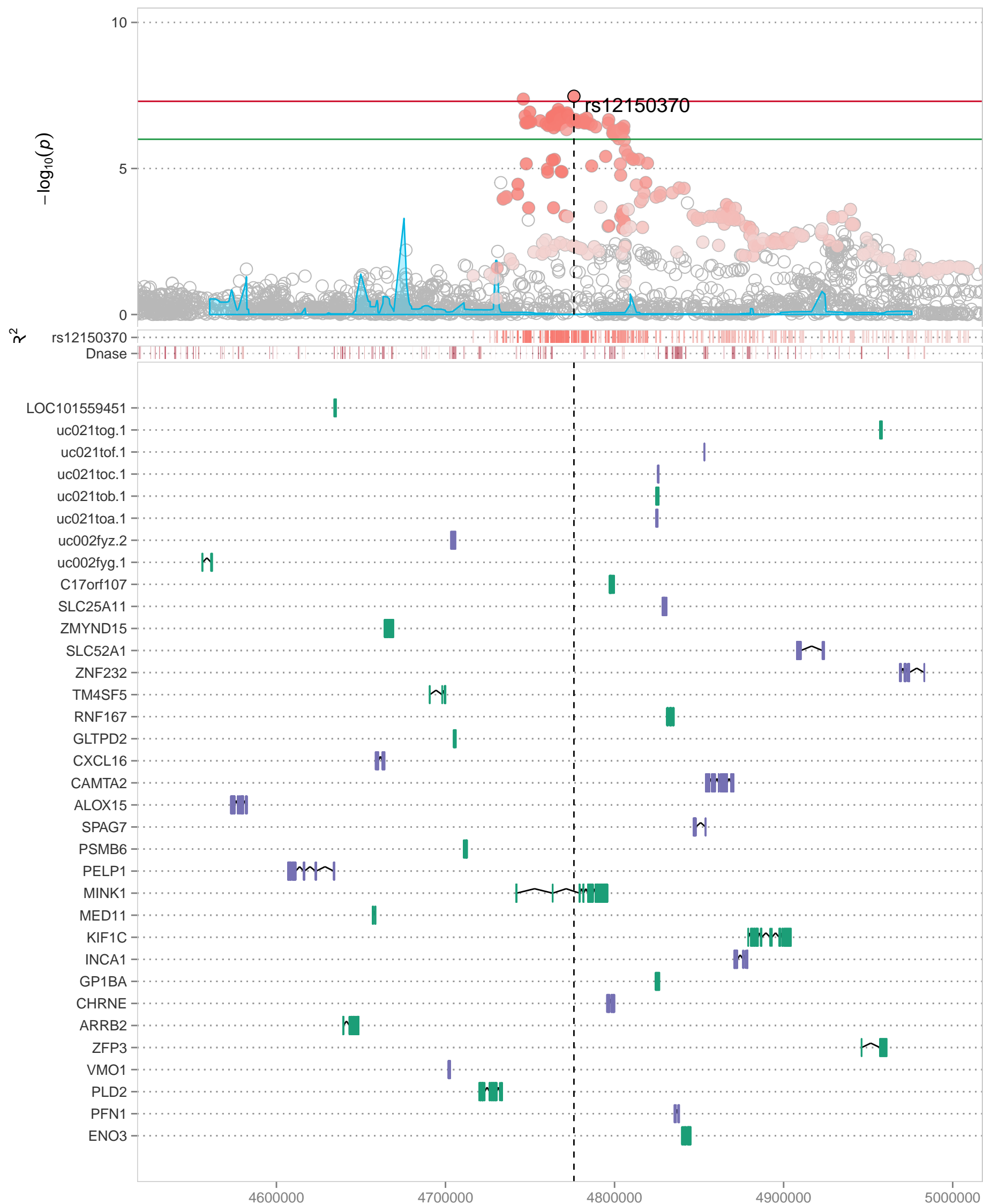

Supplement: S20 Fig — (PDF) [file pgen.1007739.s020.pdf]

Figure S21 Min SpO2 EA 2q12 rs76720903

chr2

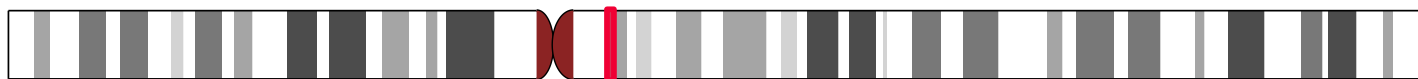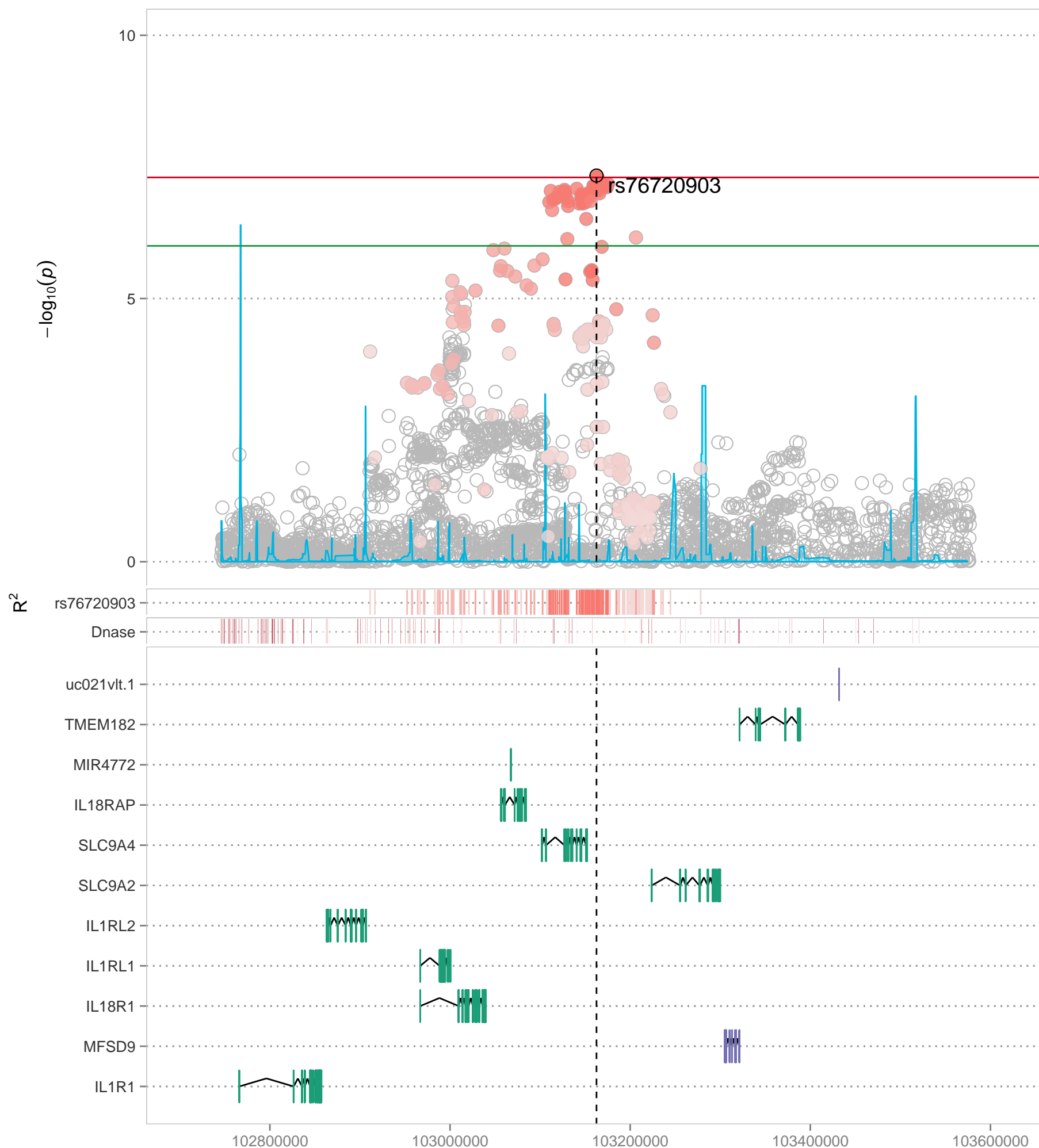

Supplement: S21 Fig — (PDF) [file pgen.1007739.s021.pdf]

Figure S22 Per90 7q22 rs2711851

chr7

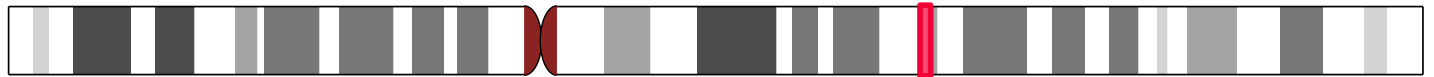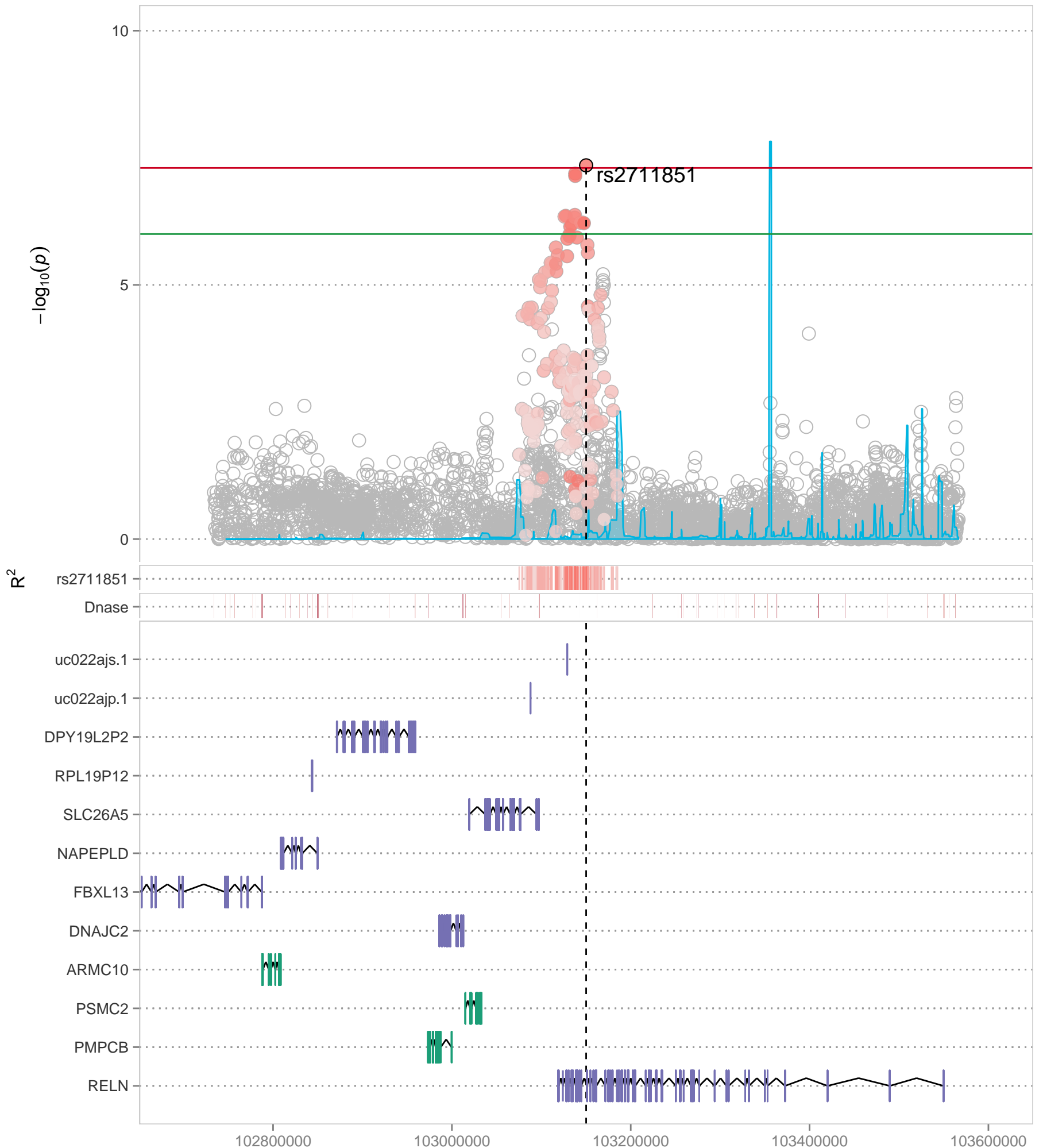

Supplement: S22 Fig — (PDF) [file pgen.1007739.s022.pdf]
